# f-scLVM: scalable and versatile factor analysis for single-cell RNA-seq

**DOI:** 10.1186/s13059-017-1334-8

**Published:** 2017-11-07

**Authors:** Florian Buettner, Naruemon Pratanwanich, Davis J. McCarthy, John C. Marioni, Oliver Stegle

**Affiliations:** 1 European Molecular Biology Laboratory, European Bioinformatics Institute (EMBL-EBI), Wellcome Genome Campus, Hinxton, Cambridge, CB10 1SD UK; 2 St Vincent’s Institute of Medical Research, 41 Victoria Parade, Fitzroy, Victoria 3065 Australia; 3 Cancer Research UK Cambridge Institute, Cambridge, UK; 4 Wellcome Trust Sanger Institute, Wellcome Genome Campus, Hinxton, Cambridge, UK; 5 European Molecular Biology Laboratory (EMBL), Genome Biology Unit, Meyerhofstr. 1, 69117 Heidelberg, Germany; 6 Current address: Helmholtz Zentrum München–German Research Center for Environmental Health, Institute of Computational Biology, Neuherberg, Germany

**Keywords:** Single-cell RNA-seq, Sparse factor analysis, Gene set annotations

## Abstract

Single-cell RNA-sequencing (scRNA-seq) allows studying heterogeneity in gene expression in large cell populations. Such heterogeneity can arise due to technical or biological factors, making decomposing sources of variation difficult. We here describe f-scLVM (factorial single-cell latent variable model), a method based on factor analysis that uses pathway annotations to guide the inference of interpretable factors underpinning the heterogeneity. Our model jointly estimates the relevance of individual factors, refines gene set annotations, and infers factors without annotation. In applications to multiple scRNA-seq datasets, we find that f-scLVM robustly decomposes scRNA-seq datasets into interpretable components, thereby facilitating the identification of novel subpopulations.

**Electronic supplementary material:**

The online version of this article (doi:10.1186/s13059-017-1334-8) contains supplementary material, which is available to authorized users.

## Background

Single-cell RNA-sequencing (scRNA-seq) is an established tool for assaying variability in gene expression levels between cells drawn from a population. Cell-to-cell differences in gene expression can be driven by both observed and unmeasured factors, including technical effects such as batch, or biological drivers including cell type-specific features, such as the stage of T-cell differentiation [1]. Importantly, such technical and biological factors can act upon the same genes [2, 3], meaning that they need to be modelled jointly to fully understand heterogeneity in scRNA-seq data.

Well-established approaches exist for handling observed factors, such as batch or experimental covariates [4, 5]. Additionally, methods based on factor analysis [6–8] and linear mixed models [9, 10] have been developed to capture unwanted variability that arises from unmeasured factors, first for conventional ensemble RNA-profiling experiments and more recently for scRNA-seq [2]. Examples of unobserved factors that can explain substantial variation in scRNA-seq include the number of detected genes in the cell (cellular detection rate) [3], expression signatures that reflect the quality of the cell [11], or the cell cycle state [2, 12]. Depending on the biological question at hand, these factors can mask other biological sources of variation, or they can themselves be of biological interest. There exist several software implementations of methods to infer and account for observed factors and unmeasured factors, including SEURAT [13], MAST [3], and scLVM [2]. Furthermore, by using informative marker gene sets, methods based on SVD and regression have also been employed to reconstruct individual cell state factors, such as the cell cycle [2, 12]. More recently, Fan et al. [14] introduced PAGODA, a PCA-based method that allows coordinated over-dispersion in specific gene sets to be identified.

However, these existing factor methods do not model errors in how gene sets are defined, and, more importantly, they independently fit individual processes and do not explicitly account for either the presence of additional unannotated biological factors or confounding sources of variation. Finally, existing factor methods were motivated by relatively small single-cell RNA-seq datasets (see Additional file 1 for a comparison to alternative factor models). Thanks to recent technological advances, it is now possible to routinely generate single-cell RNA-seq datasets containing hundreds of thousands of cells, which requires computationally efficient methods.

## Results and discussion

**Fig. 1:**
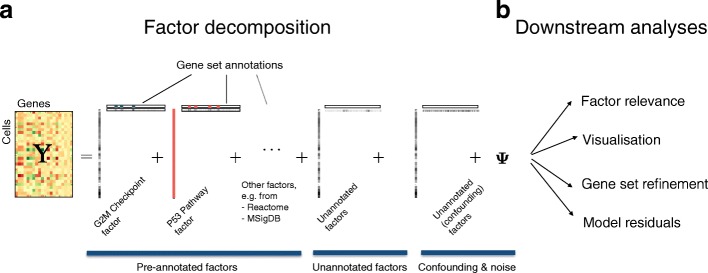
Factorial single-cell latent variable model. **a** f-scLVM is based on a variant of factor analysis, decomposing the matrix of single-cell gene expression profiles into factors and weights. Gene sets from pathway databases are used to annotate a subset of factors, with the remainder enabling the activation of unannotated factors. Unannotated factors include both *sparse*, likely biological, factors and *dense* factors, which are more likely to explain confounding sources of variation. **b** The fitted model can be used for different downstream analyses, including i) identification of biological drivers; ii) visualization of cell states; iii) data-driven adjustment of gene sets; and iv) estimation of residual expression dataset, thereby adjusting for unwanted variation and confounding effects

To address the aforementioned challenges, we here propose a factorial single-cell latent variable model (f-scLVM). Our model jointly infers factors that capture different sources of single-cell transcriptome variation, including i) variation in expression attributable to pre-annotated gene sets and ii) effects due to additional sparse factors that explain putatively meaningful biological effects. In addition to these biological factors, our model also infers likely confounding factors that are expected to affect the expression profile of the majority of genes (Fig. 1a).

To infer the state of these factors (i.e., whether a given factor is active in a cell), we employ similar assumptions as conventional factor analysis or principal component analysis (PCA). If a factor explains variation in the data, we assume that the expression levels of all genes assigned to it co-vary in a consistent manner. This allows the activity of each factor to be inferred from the data. For annotated factors, we incorporate prior annotation derived from publicly available resources such as MSigDB [15] or REACTOME [16], thereby assigning sets of biologically related genes to the same factor. The selected set of annotated factors depends on the specific question at hand and can include user-defined gene sets (“Methods”). The prior annotation is used to inform a sparse prior on the factor weights. Under this spike-and-slab prior, genes that are annotated to a factor have a higher probability of non-zero regulatory weights than other genes (“Methods”; Additional file 1). This approach allows the assignment of genes to each factor to be refined in a data-driven manner. To ensure interpretability, we also assume that only a small number of changes occur (i.e., that the initial annotation is reasonably accurate). For unannotated but biologically meaningful factors, we assume generic sparsity such that these factors drive the variation of a small number of genes. Finally, we introduce additional *dense* factors that have global effects on the expression of large numbers of genes. Similar to principles applied in population genomics [6, 7], we assume that these factors likely capture confounding effects.

As well as identifying new factors and updating existing factor annotation, our model also infers which factors explain variability in the given dataset. This factor relevance is inferred by calculating the expected variance in expression levels across cells using genes assigned to the factor. To accurately infer these variance components, f-scLVM can be used in conjunction with different observation models, thus accommodating both high-coverage datasets and sparse count profiles that are typically obtained from droplet-based experiments. Inference of model parameters, including gene assignments, factor weights, and factor states, is made using computationally efficient variational Bayesian inference, which scales linearly in the number of cells and genes. Although f-scLVM naturally builds on existing factor models, none of the existing approaches provide these features within a single model, and in particular the modelling of gene set annotations has not previously been considered (see Additional file 1 for full details and a comparison to existing factor models). The posterior distributions over model parameters facilitate a wide range of downstream analyses. These include i) the decomposition of single-cell transcriptome heterogeneity into interpretable biological drivers, ii) data visualization using factor states, iii) the refinement of gene set annotations, and iv) the estimation of a residual dataset, thereby selectively adjusting for biological or technical sources of variation (Fig. 1a, b).

### Accurate identification of gene expression drivers and gene set augmentation

**Fig. 2:**
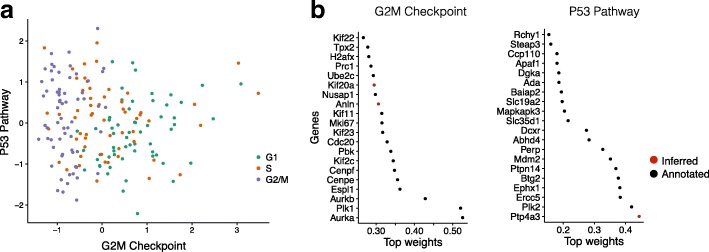
Model validation using cycling mouse embryonic stem cells. Application of f-scLVM to 182 mESCs, experimentally staged for the cell cycle. **a** Bivariate visualization of the cells using the G2M checkpoint and P53 pathway factors. The inferred G2M checkpoint factor discriminates cells in G2/M phase from the remaining cell population. **b** Weights for the most important genes in the P53 pathways and G2M checkpoint factors, showing both genes that were pre-annotated by MSIGDB (*black*) and genes added by the model (*red*)

First, to validate f-scLVM we considered a dataset where the underlying sources of variation are well understood. We applied f-scLVM to 182 mouse embryonic stem cell (mESC) transcriptomes, where each cell was experimentally staged according to its position within the cell cycle [2]. Consequently, across the entire population, we expect that the cell cycle is the major source of variation. Indeed, when applying f-scLVM using 44 core molecular pathways derived from MSigDB [15], the method robustly identified five factors, including G2/M checkpoint and P53 pathway (Additional file 2: Figure S1). These two factors could be used to stratify the cells by their position in the cell cycle (Fig. 2a). Other methods, including PAGODA, require additional post-processing steps and infer collinear and partially redundant factors that less accurately discriminated cells by cell cycle stage (Additional file 2: Figure S2), underscoring the importance of jointly modelling all annotated and unannotated factors. Furthermore, unlike existing methodology, f-scLVM enabled data driven refinement of gene set annotations (Fig. 2b). The model modified the G2/M checkpoint factor by adding two genes, Anln and Kif20a, both of which are well-characterized cell cycle regulators [17, 18]. Similarly, the model identified Ptp4a3, a known target of P53 [19], as an additional member of the P53 pathway.

**Fig. 3:**
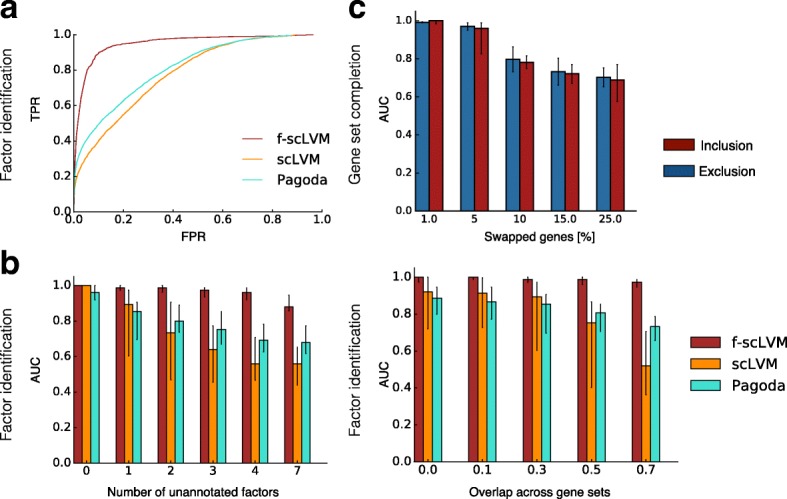
Model validation using simulated data. **a**, **b** Accuracy of f-scLVM and alternative methods for recovering the set of simulated drivers of gene expression heterogeneity. **a** Receiver operating characteristics (ROC) pooled across different simulated datasets (Additional file 2: Table S1). **b** Area under the ROC curve (*AUC*) when simulating an increasing number of unannotated factors not included in the pathway database (*left*) and when considering increasing overlap between simulated gene sets (*right*). **c** Ability of f-scLVM to augment gene sets when an increasing proportion of genes in the annotation were falsely assigned. Shown is an AUC for correctly including genes omitted from gene sets (*red*) and for removing genes that were incorrectly annotated (*blue*). Bar plots in **b**, **c** show the median AUC across 50 repeat experiments per setting with *error bars* corresponding to 25 and 75% percentiles. *FPR* false positive rate, *TPR* true positive rate

Next, we used simulations to systematically assess how robustly our model can identify relevant factors and complete gene set annotations. Over a wide range of simulations, where we varied the number of annotated factors and simulated confounders, the degree of overlap between gene sets, the number of cells, and the size of the annotated gene sets, we consistently observed that f-scLVM more accurately identified the true simulated drivers than other methods based on PCA, linear mixed models, or factor analysis (Fig. 3a; Additional file 2: Figure S3a–e). Our method showed the greatest improvement in performance over existing methods when multiple unannotated factors were simulated and when the gene sets explaining the most variance in the data contained a substantial number of overlapping genes (Fig. 3b). We also confirmed that f-scLVM was robust to extremely sparse datasets, typical of droplet-based approaches (Additional file 2: Figures S3f–h and S11).

Additionally, we considered datasets with simulated errors in gene set annotation to assess the model’s ability to adjust these gene sets appropriately (“Methods”). We observed that the model accurately identified genes that should be excluded from and added to gene sets (Fig. 3c; Additional file 2: Figure S4). Unsurprisingly, as the fraction of errors in the gene set annotation increased, the ability of the model to recover the true sets declined—however, in the more realistic setting where only a small fraction of genes were poorly annotated (1–10%), our model performed extremely well.

### Application to neuronal cells

**Fig. 4:**
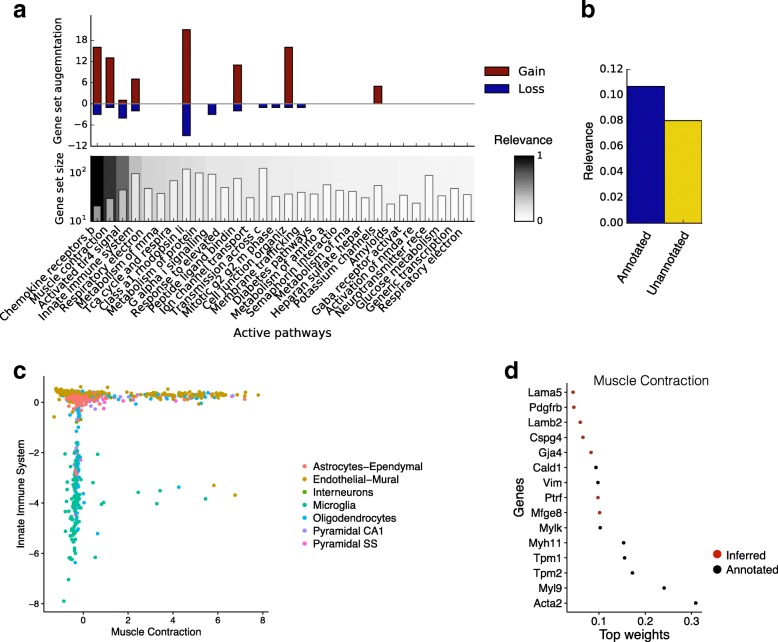
Application of f-scLVM to neuronal cells. **a** Factor relevance and gene set augmentation for the most important 30 factors identified by f-scLVM based on REACTOME pathways. *Bottom panel*: Identified factors and corresponding gene set size ordered by relevance (*white* = low relevance; *black* = high relevance). *Top panel*: Gene set augmentation, showing the number of genes added (*red*) and removed (*blue*) by the model for each factor. **b** Breakdown of the cumulative factor relevance of annotated and unannotated factors (see also Additional file 2: Figure S5). **c** Bivariate visualization of cells using the factors muscle contraction and innate immune system. Colours correspond to cell types identified in [20]; numbers denote relative factor activities inferred by the model. **d** Weights for the most important genes in the muscle contraction factor, showing both genes that were pre-annotated by REACTOME (*black*) and genes added by the model (*red*)

Having validated the performance of f-scLVM, we next applied it to a population of 3005 neuronal cells [20]. Using gene sets derived from REACTOME pathways as annotation, our model supported the importance of a set of factors similar to those identified by methods such as PAGODA (Fig. 4a), but with important differences (e.g. innate immune system; Additional file 2: Figure S5). Additionally, our model suggested a refined annotation for some of the most relevant gene sets (Fig. 4a), with, on average, 10% of genes being added and 3% of genes being removed for the top 20 annotated factors. Furthermore, the model identified unannotated factors with a high relevance score (Fig. 4b), demonstrating the importance of modelling such factors. We observed that many of these unannotated factors were sparse and captured differences between cell types that are not readily reflected in the pathway annotations (Additional file 2: Figures S5 and 6a–c).

The top ranked annotated processes separated the cells into well-defined groups, with the endothelial-mural cells being stratified into two populations by the muscle contraction factor (Fig. 4c). Notably, our model augmented the corresponding gene set by activating several genes that have previously been implicated in muscle contraction but that were not present in the pre-defined REACTOME gene set (Fig. 4d). Among the 13 identified genes were several known markers of vascular smooth muscle cells, including Rgs4, Mtfge8, and Notch3 [21–24]. A second major driver identified by f-scLVM was the innate immune system factor, which, in particular, separated microglia (nervous system immune cells) from the remaining cell types (Fig. 4c). Moreover, similar to the muscle contraction factor, the gene set was also augmented with meaningful genes (Additional file 2: Figure S6d).

In addition to the populations of neurons characterized by Zeisel et al. [20], we also applied f-scLVM to a variety of other datasets, identifying relevant factors that explained complementary axes of variation, augmenting gene sets, and observing that a considerable proportion of the variance explained could be attributed to unannotated factors (Additional file 2: Supplementary analyses and Figures SN1–3). We also assessed the robustness of the annotated factors identified by f-scLVM (Additional file 2: Figure S10).

### Scalability to datasets with tens of thousands of cells

**Fig. 5:**
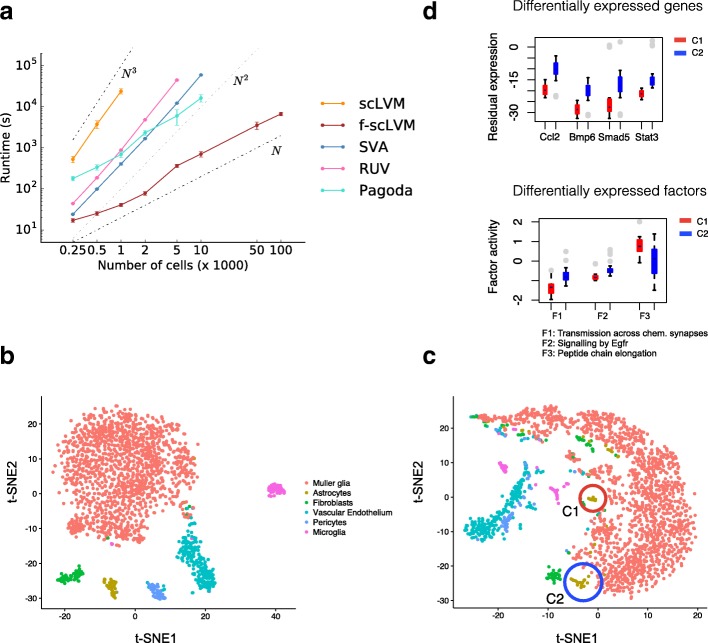
Application of f-scLVM to large-scale scRNA-seq datasets. **a** The empirical runtime when applying f-scLVM and alternative factor models (RUV, SVA, scLVM, PAGODA) to datasets with increasing size. f-scLVM scales linearly in the number of cells, enabling its use on large datasets with up to 100,000 cells. None of the existing methods could be applied to the largest dataset. *Error bars* denote plus or minus one standard deviation. **b-d** Application of f-scLVM to 49,300 retina cells profiled using Drop-Seq. **b** Visualization of a subset of 2145 cells using a non-linear t-SNE embedding. Colours correspond to cell types identified in [13]. **c** Analogous t-SNE embedding as in **b**, but on residual data (“Methods”; Additional file 2: Figure S8). The analysis on the residual dataset revealed additional substructure between cells, including two sub clusters of astrocytes (C1 and C2). **d** Genes and factors that were differentially expressed (false discovery rate < 10%) between the newly identified astrocyte clusters highlighted in **c**. The colour code is consistent with panel **c**; *grey dots* denote outlying cells. Box plots show median expression and the first and third quartile, whiskers show 1.5 × the interquartile range above and below the box

Finally, given the increasing trend to generate scRNA-seq datasets containing tens to hundreds of thousands of cells, we contrasted the computational efficiency of our model with a variety of factor analysis models (Figure 5a; Additional file 2: Figure S7). We observed that, irrespective of the number of cells, f-scLVM had a lower runtime than other approaches, with a linear scaling time in the number of cells as opposed to quadratic or even cubic relationships for other approaches. Similarly, f-scLVM has a linear time-scaling in the number of genes (Additional file 2: Figure S7). As a consequence, f-scLVM can be applied to large-scale droplet-derived datasets.

To illustrate this, we applied f-scLVM to expression profiles from 49,300 retinal cells profiled using Drop-seq [13]. Again, unannotated factors explained substantial variation in the data, where most of this variation could be attributed to a single factor that affected a large number of genes (Additional file 2: Figure S8a), suggesting that it may correspond to confounding effects. Indeed, this factor was correlated with the cellular detection rate (Additional file 2: Figure S8f), a known confounding feature of scRNA-seq datasets [3]. As previously, our model identified biologically plausible processes, including GPCR signalling and transmission across chemical synapses, as explaining variability in the data (Additional file 2: Figure S8a).

To further explore these data, we next considered the residual dataset generated after regressing out the effect of the unannotated, confounding, factor (“Methods”) [25]. When focusing on a set of six related and well defined cell types identified in the primary analysis—Müller glia, astrocytes, fibroblasts, vascular epithelium, pericytes, and microglia—the residual data revealed two subpopulations of astrocytes (Fig. 5b, c), as well as two subpopulations of microglia (Additional file 2: Figure S8b–d). In total, 1024 genes were differentially expressed between the two astrocyte populations (Wilcoxon rank sum test, false discovery rate (FDR) < 10%; Additional file 3). These were enriched for processes related to immune response and activation of astrocytes, such as inflammatory response [26], BMP signaling pathway, and cellular response to BMP [27, 28] (Additional file 2: Table S2), and included known genes related to reactive/inflammatory processes in astrocytes, such as Ccl2 [26]. In addition, BMP signalling is known to activate distinct downstream transcription factors, including Stat3 and Smad5, which show the expected pattern of behaviour between the two newly identified populations [29] (Fig. 5d). Taken together, these results show that f-scLVM can be used to infer biological and confounding factors from large datasets.

More broadly, we considered a range of available datasets and investigated the nature of unannotated factors inferred by our model. We observed, as above, that these factors were often associated with technical experimental features that have previously been suggested to underpin variability in scRNA-seq data, including the number of expressed genes and sequencing depth (Additional file 2: Figure S9). However, these associations were often weak, suggesting that the inferred hidden factors help to capture additional unwanted variation that cannot be assigned to measured covariates.

## Conclusions

Here, we have proposed a scalable factor analysis approach to comprehensively model the sources of single-cell transcriptome variability. Unique to our model is the ability to jointly infer both annotated and unannotated factors, including confounders, and to augment predefined gene sets in a data driven manner. Additionally, f-scLVM is computationally efficient, allowing analysis of very large datasets containing hundreds of thousands of cells.

We have validated our model using simulations as well as real data where the sources of transcriptome variability are well understood. Subsequently, we have applied the model to a range of different experimental settings, demonstrating its ability to infer drivers of transcriptome variation, adjust gene sets to discover new marker genes and account for hidden confounding factors in the data.

Of course, our model is not free of limitations. A general challenge for any method is to reliably differentiate confounding factors from biological signal. f-scLVM addresses this through specific assumptions on the effect of these factors (sparse versus dense) in conjunction with leveraging gene set annotation from pathway databases. Nevertheless, it is critical to interpret the model results and, in particular, the unannotated factors in the context of a given dataset.

A second potential caveat is the lack of accurate gene set annotation, which will necessarily impact the quality of the results. To mitigate this challenge, f-scLVM models possible errors in the annotation explicitly and augments gene sets in a data driven manner. However, such inferences have limitations. One important challenge is collinearities between annotated factors and true biological differences. For example, if cells in different stages of the cell cycle are systematically associated with different cell types, the results from gene set refinements may be misleading and collapse two distinct biological processes into a single factor.

Other technical aspects of the model could be improved in the future. The noise model we use at present is based on a Hurdle model [3], which could be adapted to more specifically model the noise properties of different experimental platforms. Also, our model is intrinsically linear and in particular it assumes that the inferred factors have linear additive effects on gene expression. It would be possible to model interactions between factors or different conditions, for example to assess whether a specific pathway is more active in a defined subset of cells. Extensions of f-scLVM in this direction will be an important area of future work. Finally, we note that f-scLVM could also be applied to other data modalities. In parallel to scRNA-seq, there are increasingly opportunities to model single-cell epigenome variation data, including single-cell DNA methylation profiling [30, 31], single-cell ATAC-seq [32] and multi-omic methods [33, 34].

## Methods

### The factorial single-cell latent variable model (f-scLVM)

f-scLVM is based on a variant of sparse factor analysis, decomposing the observed gene matrix into a sum of contributions from C measured covariates, A annotated factors, whose inference is guided by pathway gene sets, and H additional unannotated factors:

1 $$ \mathrm{Y}\kern0.5em =\kern0.5em \underset{\mathbf{cellcovariates}}{\underbrace{\sum \limits_{\mathrm{c}=1}^{\mathrm{C}}{\mathbf{u}}_{\mathrm{c}}\ {{\mathbf{V}}_{\mathrm{c}}}^{\boldsymbol{T}}}}\kern0.5em +\kern0.5em \underset{\mathbf{annotatedfactors}}{\underbrace{\sum \limits_{\mathrm{a}=1}^{\mathrm{A}}{\mathbf{p}}_{\mathrm{a}}\ {{\mathbf{R}}_{\mathrm{a}}}^{\boldsymbol{T}}}}\kern0.5em +\kern0.5em \underset{\mathbf{u}\mathbf{nannotatedfactors}}{\underbrace{\sum \limits_{\mathrm{h}=1}^{\mathrm{H}}{\mathbf{s}}_{\mathrm{h}}\ {{\mathbf{Q}}_{\mathrm{h}}}^{\boldsymbol{T}}}}\kern0.5em +\kern0.5em \boldsymbol{\Psi} . $$

Here, **Y** denotes the gene expression matrix where rows correspond to each of N cells and columns correspond to G genes. The vectors **u**_c_, **p**_a_, and **s**_h_ correspond to known cell covariates, as well as cell states for annotated and unannotated factors, and **V**_c_, **R**_a_, and **Q**_h_ are the corresponding regulatory weights of a given factor on all genes. The matrix **ψ** denotes residual noise, with its specific form depending on the noise model employed (see below). For the statistical derivation (see also Additional file 1), we express the model in Eq. 1 using matrix notation, collapsing the factors into a factor activation matrix **X** = [**u**_1_,.., **u**_C_, **r**_1_,.., **r**_A_, **s**_1_, …, **s**_H_] (with the comma denoting concatenation of columns), where each factor is enumerated using an indicator k = 1.. K, and K denotes the total number of fitted factors K = C + A + H. The analogous matrix representation is used for weights **W**, resulting in:

$$ \mathbf{Y}=\mathbf{X}{\mathbf{W}}^{\boldsymbol{T}}+\boldsymbol{\uppsi} . $$

Known covariates, annotated factors, and unannotated factors then correspond to different distributional assumptions on the column vectors of the matrices **X** and **W**. For brevity, we omit the cell covariates in the derivations below (Additional file 1).

We place standard multivariate normal prior distributions on the factor states of both annotated and unannotated factors. For annotated factors, we employ two levels of regularization on the corresponding columns of the weight matrix **W**. First, gene set annotations are used to inform a regulatory sparseness prior on the rows of **W** [35], thereby confining the inferred weights to the set of genes annotated in the pathway database. Second, we employ regularization of the overall variance explained by individual factors (factor relevance), thus allowing the model to deactivate factors that are not needed to explain variation in a given dataset.

#### Regulatory sparseness prior

Gene set annotations inform a spike and slab mixture prior on the elements of **W**. The regulatory weight of factor *k* on gene *g* is modelled using a Normal distribution (with factor-specific precision *α*_*k*_) if the regulatory link is active (z_*g*,*k*_ = 1), and a delta distribution otherwise to force the weights of inactive regulatory link to zero:

2 $$ p\left({w}_{g,k}|{z}_{g,k}\right)=\kern0.36em \left\{\begin{array}{cc}N\left({w}_{g,k}|0,\kern0.36em 1/{\alpha}_k\right)& \mathrm{if}\;{\mathrm{z}}_{g,k}=1\\ {}{\delta}_0\left({w}_{g,k}\right)& \mathrm{otherwise}\end{array}.\right. $$

The true state of the indicator variable *z*_*g*,*k*_, which determines whether factor k regulates gene g, is unobserved. However, pathway annotations provide partial evidence which can be used to infer the most likely state of *z*_*g*,*k*_:

$$ p\left({I}_{g,k}^n|{z}_{g,k}\right)=\left\{\begin{array}{cc}\mathrm{Bernoulli}\kern0.24em \left({I}_{g,k}^n=1\;|1-\mathrm{FPR}\right)& \mathrm{if}\kern0.24em {\mathrm{z}}_{g.k}=1\\ {}\mathrm{Bernoulli}\kern0.24em \left({I}_{g,k}^n=1\;|\kern0.24em \mathrm{FNR}\right)& \mathrm{otherwise}\end{array}.\right. $$

Here, *I*_*g*,*k*_^*n*^ is a binary variable denoting whether gene *g* is annotated to pathway *k* in the annotation database. The pathway annotation is modelled as observed data for each individual cell, thereby ensuring that the relative contribution of the annotations is independent of dataset size (Additional file 1). The rate parameter FPR corresponds to the probability of a false positive assignment and FNR denotes the probability of a false negative assignment. In the experiments, we use FNR = 0.001 and FPR = 0.01. Because the annotation information is modelled in the likelihood, the prior on the indicator variables is uninformative, *z*_*g*,*k*_ ~ Bernoulli(0.5).

During inference, the posterior distribution of the indicator variables *z*_*g*,*k*_ is then estimated jointly from the observed expression data and the annotation. Once trained, the marginal posterior estimates of *z*_*g*,*k*_ allow identification of genes that are added to or removed from a particular factor, thereby augmenting the annotation in a data-driven manner.

#### Automatic relevance determination for determining factor relevance

In addition to the regulatory sparseness prior, f-scLVM uses automatic relevance determination (ARD) [36] to regularize the variance explained by individual factors. This is achieved by placing a hierarchical prior on the precision of the normal priors for active links (Eq. 2) *α*_*k*_ ~ Γ(*a*, *b*). The precision *α*_*k*_ will be large for factors with low relevance, which corresponds to low prior variance, thus driving the regulatory weights to zero. The prior variance 1/*α*_*k*_ can also be interpreted as a measure of the regulatory impact of a factor and corresponds to the expected variance explained by the factor, for the subset of genes with a regulatory effect (see “Downstream analysis” section).

#### Modelling unannotated factors

In addition to annotated factors, f-scLVM jointly estimates the effect of a fixed number of unannotated factors. In the experiments, we consider two types of unannotated factors. First, to infer likely confounding factors, we assume that confounding factors have broad effects on large proportions of all genes, a principle that is widely used in population genomics [6, 7]. This prior belief is encoded using the Bernoulli prior *z*_*g*,*k*_ ~ Bernoulli(0.99), as a result of which the weights for these factors are effectively only regularized by the ARD prior. Second, f-scLVM allows inference of an additional set of sparse unannotated factors. These factors can, for example, be used to model additional biological variation that is not well captured by the factors in the annotation. These sparse factors are modelled using a Bernoulli prior that favours a small number of active links *z*_*g*,*k*_ ~ Bernoulli(0.01). The decision on how many factors of each type to consider can be guided by heuristics and diagnostics; see section “Diagnostics and f-scLVM parameter settings” for details on the selection of specific models.

#### Noise model

f-scLVM supports alternative noise models to accommodate different RNA-sequencing protocols. First, the standard option is the log normal noise model, where the expression matrix ***Y*** consists of log count values which are modelled assuming i.i.d. heteroscedastic residuals **ψ** (Additional file 1). We infer different residual variances for each dimension (gene), thereby accounting for varying extents of over-dispersion and enabling the model to deactivate some input dimensions, an approach widely adopted in conventional factor analysis [37].

In order to model the zero-inflation resulting from prominent dropout effects for protocols such as Drop-seq [13], f-scLVM can alternatively be run in conjunction with a zero inflation (Hurdle) noise model. A separate Bernoulli observation noise model is used, when no expression (zero count values) is observed for any specific expression value, while all remaining values are modelled using the aforementioned log Gaussian noise model. Formally, we define the factor analysis model on latent variables, F = XW^T^, and use the compound likelihood:

$$ P\left({y}_{n,g}|{f}_{n,g}\right)=\left\{\begin{array}{cc}\frac{1}{1+\mathit{\exp}\left({f}_{n,g}\right)}& if\;{y}_{n,g}=0\\ {}N\left(\mathit{\log}\left({y}_{n,g}+1\right)|{f}_{n,g},{\sigma}_g^2\right)& \mathrm{otherwise}\end{array}\right. $$

where, analogous to the log normal noise model, *y*_*n*,*g*_ corresponds to log count observations. Note that in the absence of zero counts, this noise model reduces to the basic noise model.

Finally, if zero-inflation is less likely, for example in deeply sequenced datasets with larger quantities of starting material per cell, f-scLVM can also be used in conjunction with a classic Poisson noise model. The inference approach is analogous to the dropout model; however, assuming the following likelihood model:

$$ P\left({y}_{ng}|{f}_{ng}\right)=\lambda {\left({f}_{ng}\right)}^{y_{ng}}{e}^{-\lambda \left({f}_{ng}\right)}, $$

with link function $\lambda \left({f}_{ng}\right)=\mathit{\log}\left(1+{e}^{f_{ng}}\right)$ and *y*_*n*,*g*_ now denoting raw count values.

#### Parameter inference

Closed-form inference in sparse factor analysis models such as f-scLVM is not tractable. In order to achieve scalability to large numbers of cells and genes, we employ deterministic approximate Bayesian inference based on variational methods [38]. The core idea of variational Bayes is to approximate the true posterior distribution over all unobserved variables using a factorized form. This assumption of (partial) factorization of the posterior allows derivation of an iterative inference scheme, updating posterior distributions for individual parameters in turn, given the state of all others. An important design choice in f-scLVM is to couple the approximate posterior over the regulatory sparsity indicator *z*_*g*,*k*_ and the model weights *w*_*g*,*k*_. For full details and the update equations for the f-scLVM see Additional file 1.

#### Downstream analysis

The fitted f-scLVM model facilitates a range of different downstream analyses.

##### Factor relevance

The relevance of different factors, including annotated pathway factors, is deduced from the ARD variance 1/*α*_*k*_, which corresponds to the expected explained variance of factor *k* for the subset of genes with a regulatory effect (e.g. Fig. 3a, b).

##### Visualization

The posterior distribution over the inferred factors **X** allows cell states to be visualised (e.g. Fig. 4c). This is possible for both annotated and unannotated factors. In the latter case, sparse unannotated factors frequently tend to capture additional structure between cell types (Additional file 2: Figures S5 and S6).

##### Gene set refinement

By comparing the posterior distribution on the indicator variables *z*_*g*,*k*_ with the prior gene set annotations *I*_*g*,*k*_, it is possible to identify individual genes that were added to or removed from a pathway factor during inference (e.g. Fig. 4a, d). We use the posterior threshold of 0.5 to identify genes that were added to or removed from a gene set.

##### Estimation of residual expression datasets and imputation

The learned factor **X** in combination with the corresponding regulatory weights **W** can also be used to calculate residual datasets where the effects of selected factors are removed, or to obtain imputed datasets. When using the dropout noise model, expression residuals are estimated based on the latent expression values **F** (see “Noise model” section above). In this instance the model implicitly uses the dropout noise model to impute zero values prior to estimating expression residuals (Additional file 1).

#### Relationship to other factor analysis models

Several existing factor analysis methods are related to f-scLVM. First, factor analysis with dense unannotated factors is used to adjust for unwanted variation in bulk datasets, including SVA [6], RUV [8], and PEER [7]. However, unlike f-scLVM, these methods do not model annotated factors using gene sets and hence are not designed for identifying biological drivers. Second, methods such as PAGODA [14] use gene set annotations to identify interpretable factors. However, this does not explicitly model variation outside the annotation, and it infers factors sequentially, which can lead to collinearities (Additional file 2: Figure S2b, e, f). Finally, there exist methods based on sparse factor analysis, including non-parametric methods and factor models that account for the specifics of single-cell transcriptome noise. f-scLVM is related to these variants of factor analysis, all of which are based on a linear additive model. Again, these methods do not utilize gene set annotations. f-scLVM generalizes many of these methods and, in particular, offers favourable computational efficiency. For further details and a detailed comparison of the features offered by different methods see Additional file 1: Section 2.

### Implementation of alternative methods

We compared the performance of f-scLVM to that of existing factor models. First, we ran PAGODA using the scde R package [14]. Briefly, PAGODA infers a gene-specific residual variance by deriving cell-specific error models accounting for dropout effects. This error model is then used to perform weighted PCA [39] on individual gene sets in turn, followed by a ranking of gene sets based on the explained variance of the leading PC. We also considered the single-cell latent variable model (scLVM [2]), which analogously to PAGODA infers independent low-rank factors based on predefined gene sets. The proportion of average variance explained by individual factors for the set of annotated genes, as determined using the variance decomposition described in [2], was used to rank factors. A second class of methods we compared to are factor models that do not explicitly incorporate gene set annotations. Among these we used a conventional PCA fitted to the set of all expressed genes. Second, we applied the recently proposed Zero-Inflated Factor Analysis model (ZIFA) [40], a factor analysis implementation that explicitly models dropout events. Third, we applied a sparse factor analysis model based on the Indian buffet process (IBP), a non-parametric model that automatically infers the most appropriate number of sparse factors. None of these methods annotate the inferred factors and hence we implemented a post-processing step based on a gene set enrichment (using Fisher’s exact test based on the 40% of genes with the highest absolute weights; python package xstats.enrichment) to annotate the learnt factors using the same gene sets used to fit f-scLVM. We then used the enrichment *p*-value to rank the annotated individual gene sets as potential biological drivers.

For runtime assessments, we additionally considered two approaches to account for unwanted variation. SVA timings were reported using the R implementation of the SVA package [41], considering a fixed number of surrogate variables that correspond to the true number of simulated factors. RUV runtime results were obtained using the RUV2 function from the R implementation, which estimates and adjusts for unwanted variation using control genes. Runtime estimates were obtained using the time module in python (time() function) and the proc.time() function in R; all simulations were run on eight cores of an Intel Xeon 2.60 GHz CPU.

### Datasets and pre-processing

#### Choice of the gene set annotations used in the model

In principle, large numbers of annotated factors can be inferred based on large gene set annotations. Factors that correspond to inactive pathways will be deactivated during inference. However, in practice there are trade-offs such that compact gene sets can be advantageous as the model scales computationally linearly with the number of factors. Empirically, we found that databases with tens to hundreds of annotated pathways result in a good trade-off between resolution and run-time. In particular, the MSigDB core processes database (hallmark gene sets H) consists of a comprehensive list of 50 pathways, allowing for a good resolution in relatively short runtimes. For a more fine-grained analysis, we chose the REACTOME database consisting of 674 pathways.

#### Diagnostics and f-scLVM parameter settings

By default f-scLVM is fitted using annotated factors guided by gene set annotations and additional dense unannotated factors that capture unwanted variation. However, for some datasets this set of factors may not be sufficient to explain the observed heterogeneity, e.g. because potential differences between cell types may not be well reflected by the provided annotations. In this case, it is advised to infer an additional set of sparse unannotated factors. A suitable diagnostic for this decision are excessive augmentations of the annotated gene sets such that the inferred factor is unlinked to the annotated biological process. In the software implementation of f-scLVM, sparse unannotated factors are activated if the standard model changes (gains or losses) at least 100% of annotations for at least one annotated factor. For sparse and dense unannotated factors, we considered five and three factors by default, respectively. Note that because the ARD prior allows unused factors to be deactivated, the model is robust with respect to the number of dense unannotated factors, provided a sufficiently large number is inferred (Additional file 2: Figure S3i).

#### Filtering and gene selection of single-cell RNA-seq datasets

We applied f-scLVM to datasets filtered for high-quality cells and variable genes (see also individual datasets below). Analogous to commonly applied filtering steps for t-SNE and other scRNA-seq visualization approaches, we recommend applying f-scLVM to genes that vary significantly across the set of cells. This set of genes can be identified based on ERCC spike-ins or a mean-CV relationship of endogenous genes. Genes in the tail of the variance distribution are dominated by technical sources of variation and hence can be discarded without loss of information.

#### Simulation study

We simulated gene expression matrices based on a linear additive model, an assumption that is motivated by the generative model that underlies both f-scLVM and existing alternative approaches, all of which are based on variants of linear factor analysis models (Additional file 1). We simulated effects from between three and ten active pathway factors with partially overlapping gene sets for each factor (see below), additional effects due to unknown confounding factors, and observation noise. We considered a total of 44 simulation settings, considering variable dataset sizes (cell count), variable numbers of active pathway factors, and increasing numbers of simulated unannotated confounding factors. Additionally, we varied the overlap of genes annotated to individual pathways and the size of the individual gene sets and added varying degrees of noise in the annotation provided to each respective model by simulating a certain proportion of false negative/false positive annotations and by swapping genes between active factors (see Additional file 2: Table S1 for full details of simulation parameters).

Each simulated dataset consisted of between 20 and 500 synthetic cells and 6000 genes. Gene set sizes were determined by sampling from REACTOME pathways (considering 421 pathways with 20 to 933 genes). When ranking active pathways, we compiled an annotation consisting of the true drivers and an additional set of 15 non-active pathways as a negative set, and provided it to each considered method. Confounding factors, if simulated, were generated analogously to the approach described in [10], assuming broad effects affecting between 400 and 3000 randomly selected genes. The annotations of simulated pathways were generated sequentially, ordering pathways by decreasing size and drawing genes with a selected overlap to already existing pathways. Pathways were simulated to have overlapping gene sets, between 0.0 (no overlap) and 0.7 (70% of the genes overlap). To test for the impact of the size of gene sets, we additionally considered sampling REACTOME pathways with between 20 and 50, 50 and 100, and 100 and 200 genes. To assess the robustness of f-scLVM to incorrect gene set annotations, we simulated between 1 and 50% of false negative and between 1 and 10% false positive genes in the gene set annotations of individual factors. We also considered more challenging mis-annotations by introducing gene-swaps between pairs of active factors (for between 1 and 25% of all genes). Factor activations as well as non-zero regulatory weights were drawn assuming a unit variance normal distribution. Residual noise was simulated as normally distributed with standard deviation 0.1. When dropout was simulated, we considered two alternative dropout mechanisms. First, we model a threshold effect by setting all values less than a given threshold to 0; this reflects a limit of detection where small numbers of molecules cannot be detected reliably. Second, we considered modelling the probability of dropout events as a function of the true expression level, assuming an exponential relationship [40]: *p*_*drop*_ = exp(−*λf*_*ng*_^2^), with *f*_*ng*_ being the latent expression level introduced above and *λ* the exponential decay parameter. Both dropout processes are simulated, where each setting is parameterized by *λ* and the threshold value, which corresponds to the lower limit of detection. For each simulation setting, 50 independent datasets were generated.

To assess the performance of f-scLVM and alternative methods, we consider the receiver operator characteristics (ROC) for identifying the true simulated drivers. For f-scLVM the factor relevance was used to rank factors. Analogous metrics were derived for all alternative methods (see “Implementation of alternative methods” section).

Additionally, we assessed the ability of f-scLVM to augment corrupted gene set annotations (Fig. 2c; Additional file 2: Figure S4). We evaluated the ability of the model to correct the false positive and false negative annotations separately.

#### Staged mouse embryonic stem cells

The set of 182 mESCs staged for the cell cycle have previously been described in [2]. Briefly, cells were cultured in serum-free NDiff 227 medium (Stem Cells Inc.) supplemented with 2i inhibitors and sorted by cell cycle phases (G1, S G2/M) using FACS and Hoechst staining (Hoechst 33342; Invitrogen). Cells in all three cell cycle stages were profiled using the Fluidigm C1 system. We followed the pre-processing and normalization approach as previously described [12] and considered log-transformed and size-factor adjusted (geometric library size on endogenous genes) gene expression counts for 6635 variable genes for analysis. Additional results shown in Additional file 2: Figure S1c, d were obtained when considering a size-factor normalization based on ERCC spike-ins, which retains variation in the overall amount of mRNA per cell. f-scLVM was applied using 44 gene sets derived from MSigDB (after filtering; Additional file 1). We further applied PAGODA to the raw count data of the 182 cells using the R package scde with standard settings [14].

#### Zeisel et al. dataset

We analysed log-transformed gene expression values of 3005 single neurons sequenced using a protocol with unique molecular identifier [20]. We followed the pre-processing and filtering steps from the primary publication, resulting in 7097 variable genes. f-scLVM was applied using 161 annotations from the REACTOME database (after filtering; Additional file 1), providing a high-resolution annotation. Following model diagnostics steps (see “Diagnostics and f-scLVM parameter settings”), an additional five sparse unannotated factors were added and fitted jointly with the remaining factors (Additional file 2: Figure S5). Residual datasets were generated by regressing out the effect of the most relevant unannotated factor (Additional file 2: Figure S6e–h).

#### Retina cells

We considered the normalized, log-transformed expression values of 49,300 retina cells as described in [13]. We considered all expressed genes, using the dropout noise model in f-scLVM to account for low sequence coverage. We considered gene sets from the REACTOME database. Because of the size of the data set, we used factor pre-screening to reduce the set of factors before training, retaining 50 gene sets (Additional file 1). To generate expression values corrected for confounding factors, we considered residual gene expression profiles, regressing out the effect of the most relevant unannotated dense factor. These residual data are available from [25]. Visualizations of corrected and raw expression values of six related cell types identified in the primary publication (Müller glia, astrocytes, fibroblasts, vascular epithelium, pericytes, and microglia) were obtained using t-SNE. The analysis of differentially expressed genes and factors was carried out using the Wicoxon rank sum test.

## Additional files

Additional file 1: Supplementary methods. (PDF 382 kb)

Additional file 2: Supplementary figures and tables. (PDF 14372 kb)

Additional file 3: Differentially expressed genes between the populations of astrocytes. Differentially expressed genes and factors between the identified astrocyte subpopulations (using f-scLVM residuals, Fig. 4e). (XLSX 91 kb)
