# Supplementary material for: f-scLVM: scalable and versatile factor analysis for single-cell RNA-seq
Source: Genome Biol. 2017 Nov 7;18:212. doi: 10.1186/s13059-017-1334-8 (PMC5674756; doi:10.1186/s13059-017-1334-8)
Supplement: Supplementary file 1 — Supplementary methods. (PDF 382 kb) [file 13059_2017_1334_MOESM1_ESM.pdf]

Supplementary methods

The factorial scLVM model (f-scLVM) builds on sparse factor analysis, a linear latent variable model for dimensionality reduction. In Section 1, we derive the generative model that underlies f-scLVM and present an efficient inference scheme using deterministic variational Bayesian approximations. In Section 2 we provide an overview of f-scLVM in the context of other factor analysis models. Finally, in Section 3 we discuss further details on the presented experiments, including the parameter values we use and additional robustness experiments.

1 The f-scLVM model

We here derive f-scLVM starting from the perspective of conventional factor analysis. Let $\mathbf{Y}$ by the $N \times G$ matrix of log-count expression levels for G genes observed in each of N samples (cells). We start with a bivariate linear model that factorizes the expression matrix into the sum of known covariates, annotated factors and unannotated factors:

$$\mathbf{Y} = \underbrace{\sum_{c=1}^{C} \mathbf{u}_c \mathbf{V}_c^{\mathrm{T}}}_{\text{cell covariates}} + \underbrace{\sum_{a=1}^{A} \mathbf{p}_a \mathbf{R}_a^{\mathrm{T}}}_{\text{annotated factors}} + \underbrace{\sum_{h=1}^{H} \mathbf{s}_h \mathbf{Q}_h^{\mathrm{T}}}_{\text{unannotated factors}} + \underbrace{\boldsymbol{\psi}}_{\text{residuals}} . \quad (1)$$

Here, the vectors $\mathbf{u}_c, \mathbf{p}_a$ and $\mathbf{s}_h$ are known cell covaraites, as well as facto states for annotated and unannotated factors and $\mathbf{V}_c$, $\mathbf{R}_a$ and $\mathbf{Q}_h$ are the corresponding regulatory weights of a given factor on all genes. To simplify the derivation we will collapse the factors and weights, defining $\mathbf{X} = [\mathbf{u}_1, \ldots, \mathbf{u}_C, \mathbf{r}_1, \ldots, \mathbf{r}_A, \mathbf{s}_1, \ldots, \mathbf{s}_H]$ and a corresponding concatenated weight matrix $\mathbf{W}$, resulting in

$$\mathbf{Y} = \mathbf{X} \cdot \mathbf{W}^T + \boldsymbol{\psi}. \quad (2)$$

Here, $\mathbf{W}$ denotes a $G \times K$ weight matrix that determines the regulatory affect of each factor $k \in (1, \ldots, K)$ on gene $g \in (1, \ldots, G)$. The $N \times K$ dimensional matrix $\mathbf{X}$ denotes the activity of each of $K = C + A + H$ factors in each sample and $\boldsymbol{\psi}$ is residual noise.

We start by assuming Gaussian distributed residuals, which is similar to conventional factor analysis [18] (see Section 1.3 where we discuss generalizations to count-based and dropout noise models)

$$\boldsymbol{\psi} \sim \mathcal{N}(\mathbf{0}, \text{diag}(\boldsymbol{\tau}^{-1})). \tag{3}$$

Here, $\text{diag}(\boldsymbol{\tau}^{-1})$ denotes the diagonal covariance matrix formed of the inverse elements of the noise precisions for each dimension (gene) $\boldsymbol{\tau} = (\tau_1, \dots, \tau_G)$. Together with Eq. (1) this noise model implies a Gaussian marginal likelihood of the form

$$P(\mathbf{Y}|\mathbf{W}, \mathbf{X}, \boldsymbol{\tau}) = \prod_{n=1}^{N} \mathcal{N}\left(\mathbf{y}_n \mid \mathbf{x}_n \cdot \mathbf{W}^T , \text{diag}(\boldsymbol{\tau}^{-1})\right). \tag{4}$$

We introduce a conjugate prior on the noise precisions

$$P(\boldsymbol{\tau}) = \prod_{g=1}^{G} \text{Gamma}(\tau_g \mid a_\tau, b_\tau), \tag{5}$$

where Gamma denotes the gamma distribution. The prior on the factor activities $\mathbf{X}$ is an independent normal distribution with unit variance

$$P(\mathbf{X}) = \prod_{n=1}^{N} \prod_{k=1}^{K} \mathcal{N}\left(x_{n,k} \mid 0, 1\right). \tag{6}$$

Depending on the specific choice of the prior distribution for the weight matrix $\mathbf{W}$, different factor models can be derived, including independent component analysis or conventional factor analysis [18]. We employ a structured sparsity prior that jointly models gene set annotations.

1.1 Modeling annotated and unannotated factors using a structured sparsity prior

An important difference between f-scLVM and conventional factor analysis is the two-level regularization on $\mathbf{W}$, inducing structured sparsity on the weights and thereby interpretabilty of the corresponding factors. Specifically, we first use a gene-level sparsity prior on the elements of individual columns of $\mathbf{W}$ [16, 21]. As a second level of sparseness we employ a relevance prior on the level of factors, corresponding to columns of $\mathbf{W}$, thereby deactivating factors that are unused [15].

We start by describing the structured sparsity prior for annotated factors.

1.1.1 Modeling annotated factors

Sparseness of the factors weights is encouraged via a slab and spike prior

$$P(w_{g,k} \mid z_{g,k}) = \begin{cases} \mathcal{N}\left(w_{g,k} \mid 0 , 1/\alpha_k\right) & \text{if } z_{g,k} = 1 \\ \delta_0(w_{g,k}) & \text{otherwise.} \end{cases}. \tag{7}$$

Here, $\delta_0(w_{g,k})$ denotes the Dirac delta function centered on zero (inactive links) and $1/\alpha_k$ is the prior variance of weights for active links (factor specific; see also Section 1.1.3). The indicator variable $z_{g,k}$ determines whether factor k has as a regulatory effect on gene g ($z_{g,k} = 1$) or not ($z_{g,k} = 0$).

To achieve identifiability of the fitted factors as pathways, we link the binary indicator $z_{g,k}$ to binary gene set annotations by explicitly modelling them as observed data

$$P(I^n_{g,k} \,|\, z_{g,k}) =: \rho_{g,k} = \begin{cases} \text{Bernoulli}(I^n_{g,k} = 1 \,|\, 1 - \text{FPR}) & \text{if } z_{g,k} = 1 \\ \text{Bernoulli}(I^n_{g,k} = 1 \,|\, \text{FNR}) & \text{otherwise} \end{cases}. \quad (8)$$

Here, the *observed* binary indicator $I^n_{g,k}$ determines whether gene g is annotated to a given pathway (factor) k in the annotation. The annotations are replicated such that for each sample n a complete annotation is available. Technically, this approach is equivalent to scaling the likelihood component of the annotation with the number of cells. Since the likelihood component that links the indicator $z_{g,k}$ to observed expression data (Eq. (7)) scales with the number of cells, this ensures that the relative contribution of the annotations is independent of dataset size.
The rate parameter FPR corresponds to the probability of false-positive annotations in the annotation and FNR denotes the probability of false-negative assignments.

Finally, for annotated factors the indicator variables $z_{g,k}$ are *a priori* Bernoulli distributed

$$P(z_{g,k}) = \text{Bernoulli}(z_{g,k} \,|\, \pi). \quad (9)$$

For annotated factors the sparseness structure is determined by the annotation and hence we choose an uninformative prior $\pi = 0.5$. The joint probability of all observed and unobserved data then follows as

$$P(\mathbf{Y}, \mathbf{I}, \mathbf{X}, \mathbf{W}, \mathbf{Z}, \boldsymbol{\tau}) = \prod_{n=1}^{N} \mathcal{N}\left(\mathbf{y}_n \,|\, \mathbf{x}_n \cdot \mathbf{W}^T, \; \text{diag}(\boldsymbol{\tau}^{-1})\right) \prod_{g,k} p(w_{g,k} \,|\, z_{g,k}) P(I^n_{g,k} \,|\, z_{g,k}) P(z_{g,k}) P(\tau_g). \quad (10)$$

Here $I^n_{g,k}$ is identical for all cell copies, i.e. $I^n_{g,k} = I_{g,k} \forall n$. A graphical model representation of the full model is shown in Figure M1.

1.1.2 Modelling unannotated factors and known covariates

The effect of factors that are not included in the pathway annotation is modeled within the same framework. For unannotated factors, there exists no prior information, which means that the likelihood component for the annotation prior (Eq. (8)) is omitted. The prior probability of a regulatory effect for unannated factors π determines the expected sparseness level (see Eq. (9)). In the experiments, we consider two different types of factors. For sparse factors we set $\pi = 0.1$, which corresponds to the belief that 10% of the genes are regulated by these factors. Additionally, we model dense factors at a sparseness level of 0.99 ($\pi = 0.99$). Sparse factors tend to explain biological variation that is not well captured by the pre-annotated gene sets, whereas dense factors frequently correspond to confounding factors. These principles of sparse versus dense effects are similar to ideas that have previously been considered in population genomics [14, 22].
For details and guidelines on how to set model parameters for learning unannotated factors, see Section 3.3.
Finally, cell covariates (e.g. the number of expressed genes [7], size factors, etc.) are treated analogously to dense factors. However, importantly their factor states $\mathbf{x}_k$ are observed and do not need to be inferred during training.

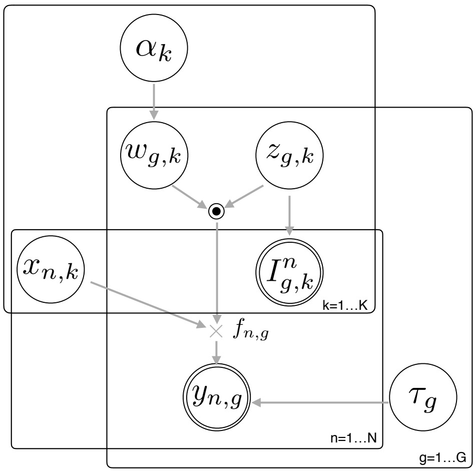

Supp. Meth. Figure M1: **Graphical model representation of f-scLVM .** Circled variables are random and unobserved. Double-circled variables denote observed data, including gene expression profiles $y_{n,g}$ and the annotation data $I^n_{g,k}$. Statistical dependencies between all variables are indicated using arrows. The filled circles linking $w_{z,k}$ and $z_{g,k}$ denote the sparsity likelihood. For simplicity we have omitted an explicit representation of unannotated factors and cell covariates, for which no prior annotations are provided.

1.1.3 Automatic relevance determination for identifying relevant factors

A conventional spike and slab prior (Eq. (7)) is based on the assumption that each factor explains the same prior variance, although the set of active genes may differ between factors.

In particular for large annotations with hundreds of factors this assumption is likely false, as only a subset of the pathways will be active in a given dataset. To model different overall regulatory importance across factors we use a second regularization based on the automatic relevance determination (ARD) prior [15]. The ARD prior has been shown to be effective for shrinking factors with low relevance, for example in the context of conventional FA models (e.g. [22, 6]). This factor level regularization is achieved by placing a hierarchical Gamma prior on the precision parameters of the regulatory prior for each factor k (see Eq. (7))

$$P(\alpha_k \mid a_\alpha, b_\alpha) \quad = \quad \text{Gamma}(\alpha_k \mid a_\alpha, b_\alpha). \tag{11}$$

For factors that do not explain variation in the data the precision α_k will be large, which corresponds to a small prior variance for the corresponding factor weights. The posterior distribution over the relevance parameters α_k can also be used to deduce the importance of individual factors; see Section 3.6.

1.2 Parameter inference

Closed-form inference in sparse factor analysis is not tractable and hence, in general, computationally expensive Monte Carlo simulations or other approximate inference schemes are required. To enable the application of f-scLVM to large datasets with up to hundreds of thousands of cells and genome-wide expression counts, we here use efficient deterministic approximations instead of Monte Carlo schemes. This fully-factorized Variational Bayesian approximation scales linearly in the number of cells and genes, which renders the applications to larger datasets feasible.

Briefly, in variational Bayesian inference, the true intractable posterior distribution of the latent (unobserved) model parameters $\mathrm{P}(\mathcal{H}\,|\,\mathcal{D})$ is approximated by a simpler (partially) factorized form $Q(\mathcal{H}) = \prod_i Q(\mathcal{H}_i\,|\,\boldsymbol{\theta}_i)$. Here, $\mathcal{D}$ denotes the observed data and $\boldsymbol{\theta}_i$ are variational parameters that parametrize the distribution of variation factors $Q(\mathcal{H}_i\,|\,\boldsymbol{\theta}_i)$.

The objective of variational Bayesian inference is to determine variational parameters $\boldsymbol{\theta}_i$ such that the Kullback-Leibler (KL) divergence between the true posterior $P(\mathcal{H}\,|\,\mathcal{D})$ and the variational approximation $Q(\mathcal{H})$ is minimized. The use of the KL divergence as a measure of distributional distance lends itself to an iterative algorithm for updating the variational parameters of individual factors sequentially. Under this approximation the log marginal likelihood is then bounded by

$$\mathcal{F} = \int \prod_i Q(\mathcal{H}_i\,|\,\boldsymbol{\theta}_i) \log \frac{P(\mathcal{H}\,|\,\mathcal{D})}{Q(\mathcal{H}_i\,|\,\boldsymbol{\theta}_i)} d\mathcal{H}_i. \tag{12}$$

This algorithm is guaranteed to minimize the KL divergence in each iteration and generalizes the widely used Expectation Maximization algorithm. For a comprehensive overview of Variational Bayesian approximate inference, see for example [5]. In each iteration, the parameters of individual variational factors $\boldsymbol{\theta}_i$ are updated in turn, given the current state of all other factors [11, 2, 3]. For any chosen factorization $Q(\mathcal{H}) = \Pi_i Q(\mathcal{H}_i\,|\,\boldsymbol{\theta}_i)$, it can be shown that the optimal update for each factor can be obtained from the average log likelihood under all other Q-distributions

$$Q(\mathcal{H}_i\,|\,\boldsymbol{\theta}_i) \propto \exp(\langle \log P(\mathcal{H})\rangle_{Q\backslash\mathcal{H}_i}). \tag{13}$$

These updates of individual Q-distributions are performed sequentially, until convergence is reached. If the chosen factorization matches the prior factorization of the model, it can be shown that the step in Eq. (13) corresponds to updating the variational parameters $\boldsymbol{\theta}_i$, whereas the functional form of the approximate Q-distribution remains in the same class as the corresponding prior distributions. For brevity, we will in the following omit the explicit dependency of each variational factor on the respective parameters $\boldsymbol{\theta}_i$.

Variational factorization of the model The first step to derive a variational inference algorithm for f-scLVM is to re-parameterize the model without the Dirac function (Eq. (7)). To this end, the elements $w_{g,k}$ are modeled as an (element wise) product of a Bernoulli random variable $z_{g,k}$ and a Gaussian random variable $\tilde{w}_{g,k}$ [25]

$$\begin{aligned} P(\tilde{w}_{g,k}\,|\,\alpha_k) &= \mathcal{N}(\tilde{w}_{g,k}\,|\;0\;,\;1/\alpha_k) \\ P(z_{g,k}\,|\,\pi) &= \text{Bernoulli}(z_{g,k}\,|\,\pi). \end{aligned} \tag{14}$$

The joint prior distribution of these two new random variables follows as

$$P(\tilde{w}_{g,k}, z_{g,k}) = \mathcal{N}(\tilde{w}_{g,k} \mid 0 \, , \, 1/\alpha_k) \, \pi^{z_{g,k}} (1-\pi)^{1-z_{g,k}}. \tag{15}$$

This re-parameterization then allows us to define a suitable factorization of the unobserved variables $\tilde{\mathbf{W}}$, $\mathbf{Z}$, $\mathbf{X}$, $\boldsymbol{\tau}$ and $\boldsymbol{\alpha}$. Variational inference is most efficient if the Q distribution is factorized, which implies independence assumptions on the approximate posterior. Such approximations are generally problematic for strongly coupled parameters. In f-scLVM this concern applies in particular to the regulatory weights $\tilde{\mathbf{W}}$ and the binary indicator variables $\mathbf{Z}$. While a factorizing assumptions, i.e. $Q(\tilde{\mathbf{W}}, \mathbf{Z}) = Q(\tilde{\mathbf{W}})Q(\mathbf{Z})$ has been considered elsewhere [25, 24], such an approach will lead to poor convergence, as the true factor with 2^K modes is approximated by a single unimodal factor [13]. In other words, two highly coupled random variables ($\tilde{\mathbf{W}}$ and $\mathbf{Z}$) are inferred assuming approximate independence, which leads to poor results. To circumvent this, we here adapt the scheme proposed by Lazaro-Gredilla & Tsitas (2011) [13], who derived an efficient and accurate variational inference for the spike and slab prior, however in the context of Multi-Task and Multiple Kernel Learning. Briefly, the key idea is to treat each pair $\{\tilde{w}_{g,k}, z_{g,k}\}$ as a single unit, choosing a joint factorization of the form $Q(\tilde{\mathbf{W}}, \mathbf{Z}) = \prod_k \prod_g Q(\tilde{w}_{g,k}, z_{g,k})$. This approach yields an approximate marginal distribution with 2^K components, which better captures the multinomial posterior distribution of $\mathbf{W}$. For all remaining model parameters we choose a fully factorized variational distribution, which delivers an overall linear runtime complexity of f-scLVM. The full approximate variational distribution follows as

$$\begin{aligned} Q(\tilde{\mathbf{W}}, \mathbf{X}, \boldsymbol{\tau}, \boldsymbol{\alpha}) &= Q(\tilde{\mathbf{W}}, \mathbf{Z}) Q(\mathbf{X}) Q(\boldsymbol{\tau}) Q(\boldsymbol{\alpha}) \\ &= \left(\prod_{g=1}^{G} \prod_{k=1}^{K} Q(\tilde{w}_{g,k}, z_{g,k}) \right) \left(\prod_{n=1}^{N} \prod_{k=1}^{K} Q(x_{n,k}) Q(\alpha_k) \right) \left(\prod_{g=1}^{G} Q(\tau_g) \right). \end{aligned} \tag{16}$$

The corresponding variational lower bound $\mathcal{F}$ of this model can be written as:

$$\begin{aligned} \mathcal{F} = \; & \langle \log P(\mathbf{Y} \mid \mathbf{X}, \tilde{\mathbf{W}}, \mathbf{Z}, \boldsymbol{\alpha}, \boldsymbol{\tau}) \rangle_{Q(\tilde{\mathbf{W}}, \mathbf{Z}) Q(\mathbf{X}) Q(\boldsymbol{\tau}) Q(\boldsymbol{\alpha})} \\ & - \langle \log P(\mathbf{I} \mid \mathbf{Z} \rangle_{Q(\tilde{\mathbf{W}}, \mathbf{Z})} \\ & - KL(Q(\mathbf{X}) || P(\mathbf{X})) - \langle KL(Q((\tilde{\mathbf{W}}, \mathbf{Z}) || P(\tilde{\mathbf{W}}, \mathbf{Z} \mid \boldsymbol{\alpha})) \rangle_{Q(\boldsymbol{\alpha})} \\ & - KL(Q(\boldsymbol{\alpha}) || P(\boldsymbol{\alpha} \mid a_\alpha, b_\alpha)) - KL(Q(\boldsymbol{\tau}) || P(\boldsymbol{\tau} \mid a_\tau, b_\tau)), \end{aligned} \tag{17}$$

with $\langle\rangle_{Q()}$ denoting the expectation under the Q-distributions $Q()$.

1.2.1 Variational update equations

Sequential updated equations for individual factors are calculated using the expectation under all remaining factors using Eq. (13). We start with the joint variational distribution for $Q(\tilde{w}_{g,k}, z_{g,k})$, which we rewrite by explicitly conditioning on the binary indicator $z_{g,k}$

$$Q(\tilde{w}_{g,k}, z_{g,k}) = Q(\tilde{w}_{g,k} \mid z_{g,k}) Q(z_{g,k}). \tag{18}$$

This allows decomposing $Q(\tilde{w}_{g,k}, z_{g,k})$ into $Q(z_{g,k})$ and the Q distribution for the corresponding weight, conditional on $z_{g,k}$. Both Q distributions retain the functional form of their respective priors (i.e. Bernoulli and Normal; Eq.(14)-(15))

$$Q(z_{g,k} = 1) = \frac{1}{1 + \exp(-u_{g,k})} = \gamma_{g,k}. \tag{19}$$

The Q distribution for the weight conditioned on $z_{g,k}$ follows as:

$$Q(\tilde{w}_{g,k} \mid z_{g,k} = 0) = \mathcal{N}\left(\tilde{w}_{g,k} \mid 0 \ , \ \alpha_k^{-1}\right) \tag{20}$$

$$Q(\tilde{w}_{g,k} \mid z_{g,k} = 1) = \mathcal{N}\left(\tilde{w}_{g,k} \mid \mu_{\tilde{w}_{g,k}} \ , \ \sigma^2_{\tilde{w}_{g,k}}\right), \tag{21}$$

with variational parameters $(\gamma_{g,k}, \ \mu_{\tilde{w}_{g,k}}, \ \sigma_{\tilde{w}_{g,k}})$. Furthermore this decomposition also allows us to re-write the second term in Eq. 17 as $\langle \log P(\mathbf{I} \mid \mathbf{Z}) \rangle_{Q(\tilde{\mathbf{W}},\mathbf{Z})} = \langle \log P(\mathbf{I} \mid \mathbf{Z}) \rangle_{Q(\mathbf{Z})}$. Update equations for these variational parameter are given below, where $\langle \rangle$ denotes the expectation under all remaining Q distributions.

$$\gamma_{g,k} = \frac{1}{1 + \exp(-u_{g,k})} \quad \text{with} \tag{22}$$

$$u_{g,k} = \log \frac{\pi}{1-\pi} + \sum_{n=1}^{N} \log \frac{\rho_{g,k}}{1-\rho_{g,k}} + 0.5 \log \frac{\langle \alpha_k \rangle}{\langle \tau_g \rangle} - 0.5 \log \left(\sum_{n=1}^{N} \langle x_{n,k}^2 \rangle + \frac{\langle \alpha_k \rangle}{\langle \tau_g \rangle} \right) + \frac{\langle \tau_g \rangle}{2} \frac{\left(\sum_{n=1}^{N} y_{n,g} \langle x_{n,k} \rangle - \sum_{m \neq k} \langle z_{g,m} \tilde{w}_{g,m} \rangle \sum_{n=1}^{N} \langle x_{n,k} \rangle \langle x_{n,k} \rangle \right)^2}{\sum_{n=1}^{N} \langle x_{n,k}^2 \rangle + \frac{\langle \alpha_k \rangle}{\langle \tau_g \rangle}} \tag{23}$$

$$\mu_{\tilde{w}_{g,k}} = \frac{\sum_{n=1}^{N} y_{n,g} \langle x_{n,k} \rangle - \sum_{m \neq k} \langle z_{g,m} \tilde{w}_{g,m} \rangle \sum_{n=1}^{N} \langle x_{n,k} \rangle \langle x_{n,k} \rangle}{\sum_{n=1}^{N} \langle x_{n,k}^2 \rangle + \frac{\langle \alpha_k \rangle}{\langle \tau_g \rangle}} \tag{24}$$

$$\sigma^2_{\tilde{w}_{g,k}} = \frac{\langle \tau_g \rangle^{-1}}{\sum_{n=1}^{N} \langle x_{n,k}^2 \rangle + \frac{\langle \alpha_k \rangle}{\langle \tau_g \rangle}} \tag{25}$$

Taken together, this means that we can update $Q(\tilde{w}_{g,k}, z_{g,k})$ using

$$Q(\tilde{w}_{g,k} | z_{g,k}) Q(z_{g,k}) = \mathcal{N}\left(\tilde{w}_{g,k} \mid \mu_{\tilde{w}_{g,k}} z_{g,k} \ , \ z_{g,k} \sigma^2_{\tilde{w}_{g,k}} + (1 - z_{g,k}) \alpha_k^{-1}\right) \gamma_{g,k}^{z_{g,k}} (1 - \gamma_{g,k})^{1 - z_{g,k}}. \tag{26}$$

Consequently, the expectation of $(\tilde{w}_{g,k} z_{g,k})$ under its Q distribution can simply be written as $\langle \tilde{w}_{g,k} z_{g,k} \rangle_Q = \gamma_{g,k} \mu_{\tilde{w}_{g,k}}$.

For the remaining variational factors, we can use standard update equations for a conventional variational factor analysis model, e.g. [9, 22]. The approximate posterior distribution for the factor activations $\mathbf{X}$ (c.f. Eq. (6)) follows as

$$Q(\mathbf{X}) = \prod_{k=1}^{K} \prod_{n=1}^{N} Q(x_{n,k}) = \mathcal{N}\left(x_{n,k} \mid \mu_{x_{n,k}} \ , \ \sigma^2_{x_{n,k}}\right), \tag{27}$$

with variational parameters $(\mu_{x_{n,k}}, \sigma^2_{x_{n,k}})$. The corresponding update equations for the variational parameters are:

$$\sigma^2_{x_{n,k}} = \left(\sum_{g=1}^{G} \frac{\langle z_{g,k} \tilde{w}^2_{g,k} \rangle}{\tau_g^{-1}} + 1 \right)^{-1} \tag{28}$$

$$\mu_{x_{n,k}} = \sigma^2_{x_{n,k}} \sum_{g=1}^{G} \langle z_{g,k} \tilde{w}_{g,k} \rangle \tau_g \left(y_{n,g} - \sum_{m \neq k} \langle z_{g,k} \tilde{w}_{g,k} \rangle \langle x_{n,k} \rangle \right). \tag{29}$$

Similarly, the Q-distribution of the ARD factor relevance parameters $\boldsymbol{\alpha}$ have the same functional form as their Gamma prior (Eq. (11))

$$Q(\boldsymbol{\alpha}) = \prod_{k=1}^{K} Q(\alpha_k) = \prod_{k=1}^{K} \text{Gamma}(\alpha_k \mid \hat{a}_{\alpha_k}, \hat{b}_{\alpha_k}), \tag{30}$$

with variational parameters $(\hat{a}_{\alpha_k}, \hat{b}_{\alpha_k})$ and update equations

$$\hat{a}_{\alpha_k} = a_{\alpha_k} + \frac{\sum_{g=1}^{G} \gamma_{g,k}}{2} \tag{31}$$

$$\hat{b}_{\alpha_k} = b_{\alpha_k} + \frac{\sum_{g=1}^{G} \gamma_{g,k} \left(\mu^2_{\tilde{w}_{g,k}} + \sigma^2_{\tilde{w}_{g,k}} \right)}{2}. \tag{32}$$

Similarly, the Q-distribution of the noise precision values $\boldsymbol{\tau}$ can be written as

$$Q(\boldsymbol{\tau}) = \prod_{g=1}^{G} Q(\tau_g) = \prod_{g=1}^{G} \text{Gamma}(\tau_g | \hat{a}_{\tau_g}, \hat{b}_{\tau_g}), \tag{33}$$

with variational parameters $(\hat{a}_{\tau_g}, \hat{b}_{\tau_g})$. The associated update equations follow as:

$$\hat{a}_{\tau_g} = a_{\tau_g} + \frac{N}{2} \tag{34}$$

$$\hat{b}_{\tau_k} = b_{\tau_k} + \frac{1}{2} \sum_{n=1}^{N} \langle (y_{g,n} - \sum_{k} z_{g,k} \tilde{w}_{g,k} x_{n,k})^2 \rangle. \tag{35}$$

1.3 Non-Gaussian noise models for (low-coverage) sequence data

The model presented so far assumes Gaussian distributed residuals. In order to appropriately account for zero inflation, a consequence of dropout effects in sparsely sequenced single-cell data, f-scLVM can also be used in conjunction with a Hurdle noise model, which explicitly accounts for dropout.

This is achieved by introducing a separate Bernoulli observation noise model for the subset of observations with zero counts in the expression matrix. For all remaining observations, the standard Gaussian noise model on a logarithmic scale is retained (see also Section 3). More formally, we introduce the matrix

factorization model on latent variables $\mathbf{F} = \mathbf{X}\mathbf{W}^T = [f_{n,g}]$, which is coupled to the observed expression count data $\mathbf{Y}$ using the likelihood model:

$$P(y_{n,g}|f_{n,g}) = \begin{cases} 1/(1+\exp(f_{n,g})) & \text{if } y_{n,g} = 0 \\ \mathcal{N}(y_{n,g} \mid f_{n,g}, 1/\kappa_g) & \text{otherwise} \end{cases}. \quad (36)$$

To achieve efficient inference in conjunction with this non-Gaussian likelihood model, we adapt prior work by Seeger et al. [20], who have proposed using local variational bounds for non-Gaussian likelihood functions in a different context. Briefly, additional variational parameters $\mathbf{\Xi} = Q(\mathbf{X})Q(\mathbf{W})^T = [\xi_{n,g}]$ are introduced which determine pseudo-observations $\tilde{\mathbf{Y}}$ based on the zero inflated data $\mathbf{Y}$, which in turn are modelled using a Gaussian noise model with precision κ_g. In the following we outline how the update equations for these pseudo-observations and κ_g can be derived.

Let $g(f_{n,g}) = -\log(P(y_{n,g}|f_{n,g}))$. If $g(f_{n,g})$ is twice differentiable and bounded by κ_g such that $g''(f_{n,g}) < \kappa_g \;\; \forall n, g$ we can use a Taylor expansion to approximate $g(f_{n,g})$

$$g(f_{n,g}) \leq \kappa_g/2(f_{n,g} + \xi_{n,g})^2 + g'(\xi_{n,g})(f_{n,g} - \xi_{n,g}) + g(\xi_{n,g}) =: q_{n,g}(f_{n,g}, \xi_{n,g}). \quad (37)$$

For non-zero observations with Gaussian noise model, this approximation is exact since $g''(f_{n,g}) = \kappa_g$ for all non-zero observations. For the Bernoulli noise model the Taylor approximation holds for $\kappa = 1/4$ since $g''(f_{n,g}) < 1/4$ for all zero observations.

We then further follow [20] and update $\mathbf{\Xi} = Q(\mathbf{X})Q(\mathbf{W})^T = [\xi_{n,g}]$ with $Q(\mathbf{W})$ and $Q(\mathbf{X})$ denoting the Q-distributions of weights and latent variables as before. In order to derive the update equations for $Q(\mathbf{W})Q(\mathbf{X})$ we bring the Taylor approximation of $g(f_{n,g})$, $q_{n,g}$, in a quadratic form and note that

$$q_{n,g}(f_{n,g}, \xi_{n,g}) \propto \kappa_g/2(f_{n,g} - (\xi_{n,g} - g(\xi_{n,g}))/\kappa_g)^2 =: -\log \mathcal{N}(\tilde{y}_{n,g} \mid f_{n,g}, 1/\kappa_g) \quad (38)$$

with $\tilde{y}_{n,g} = \xi_{n,g} - g'_{n,g}(\xi_{n,g})/\kappa_g$. Consequently, for fixed $[\xi_{n,g}]$, the update of $Q(\mathbf{X})Q(\mathbf{W})$ is equivalent to f-scLVM with pseudo-data $\tilde{\mathbf{Y}} = [\tilde{y}_{n,g}]$ and noise precision κ_g.

When using the dropout-noise model, we can thus derive updates for the pseudo-observations as

$$\tilde{y}_{n,g} = \begin{cases} \xi_{n,g} - \kappa_g/(1+\exp(f_{n,g})) & \text{if } y_{n,g} = 0 \\ y_{n,g} & \text{otherwise} \end{cases}. \quad (39)$$

Note that the pseudo-observations equal the observations $\mathbf{Y}$ for non-zero expression values.

We update κ_g - which corresponds to τ_g in the case of a Gaussian noise model - using Eq.(33)-(35), using only cells with non-zero expression values

$$\kappa_g = max(0.25, \tau_g). \quad (40)$$

Specifically, $\hat{a}_{\tau_g} = a_{\tau_g} + \frac{|N_g|}{2}$ and $\hat{b}_{\tau_k} = b_{\tau_k} + \frac{1}{2}\sum_{n \in N_g} \langle (y_{gn} - \sum_k z_{g,k}\tilde{w}_{g,k}x_{nk})^2 \rangle$, where N_g corresponds to the number of cells with observed expression values for gene g.

The updates for $\tilde{\mathbf{W}}$, $\mathbf{X}$ and $\boldsymbol{\alpha}$ are implemented as described in Section 1.2.1, however with pseudo-observations instead of $\mathbf{Y}$. This allows allows us to iteratively update the pseudo-observations $\tilde{\mathbf{Y}}$ based

on $\boldsymbol{\Xi} = Q(\mathbf{X})Q(\mathbf{W})^T$ as well as $Q(\mathbf{X})$ and $Q(\mathbf{W})$ using $\tilde{\mathbf{Y}}$. The variational parameter update for the ARD prior to identify relevant factors and the spike-and-slab prior to regularize pathway components are unchanged. This extends the approach suggested in [20] as we allow for different forms of observation noise $g_{n,g}$ as well as gene-specific precision κ_g, reflecting that the variance varies highly between genes and perform inference for κ_g.

1.3.1 Poisson noise model for count data

The analogous modeling strategy can also be employed to model count observations using a Poisson noise model. Again, variational parameters $\xi_{n,g}$ are introduced which determine pseudo-observations $\tilde{\mathbf{Y}}$ based on the observed count data $\mathbf{Y}$, which in turn are modeled using a Gaussian noise model. In contrast to the dropout-noise model, $\mathbf{Y}$ now correspond to the raw count data. More specifically, we write the Poisson likelihood with link function λ as

$$P(y_{n,g}|f_{n,g}) = \lambda(f_{n,g})^{y}_{n,g}\mathrm{e}^{-\lambda(f_{n,g})}. \tag{41}$$

As before, $g(f_{n,g}) = -\log(P(y_{n,g} \mid f_{n,g}))$ needs to be twice differentiable and bounded. We therefore choose a gene-specific link function $\lambda_g(f_{n,g}) = \log(1 + \exp(f_{n,g}))$, resulting in an upper bound of the second derivative

$$\omega_g = 1/4 + 0.17 y_{max_g}, \quad y_{max_g} = \max(\mathbf{y}_g). \tag{42}$$

We then update the pseudo-observations $\tilde{\mathbf{Y}}$ as $\tilde{y}_{n,g} = \xi_{n,g} - g'_{n,g}(\xi_{n,g})/\omega_g$ with $\xi_{n,g}$ as defined above

$$\tilde{y}_{n,g} = \xi_{n,g} - \frac{\pi(f_{n,g})(1 - y_{n,g}/\lambda(f_{n,g}))}{\omega_g}, \quad \pi(f_{n,g}) = 1/(1 + \exp(f_{n,g})). \tag{43}$$

Analogously to the dropout noise-model, updates for $\tilde{\mathbf{W}}$, $\mathbf{X}$ and $\boldsymbol{\alpha}$ are performed as described in Section 1.2.1, with pseudo-observations instead of $\mathbf{Y}$. However, unlike the Hurdle noise model there is no need to infer ω_g (corresponding to κ_g) as it is determined by Eq. (42).

2 Relationship to existing factor analysis models

The f-scLVM model is related to a number of existing variants of factor analysis, all of which are based on a linear additive model. These methods can be broadly grouped into parametric and non-parametric approaches. Non-parametric methods [12] infer the number of active factors, in principle allowing an *infinite* number of factors to be used in the model. In contrast, parametric models need the user to specify the number of latent factors before inference. One strategy to mitigate the need to specify the precise number of factors in the model is the use of an ARD prior, which was first applied in the context of probabilistic principal component analysis [4] and later for factor analysis models, including PEER [22]. This approach is also applied in f-scLVM, where a much larger number of annotated and unannotated factors are included in the model and the ARD prior deactivates unused ones.

A second aspect of regularization in factor analysis are sparsity priors to encourage element-wise sparseness of the factor loadings. f-scLVM employs a spike and slab prior, which has previously been used to achieve sparsity for this purpose, e.g. [8]. Our model additionally uses prior annotations to inform this

Table 1: Feature comparison of alternative factor models related to f-scLVM. IBP=Indian Buffet Process, TBP=Three parameter Beta Prior, EM=Expectation Maximisation, VB=Variational Bayes.

Method	Feature				
	Elementwise sparsity	Annotations	Noise model	Model selection	Inference
PCA [23]	–	–	homoscesdatic	None/ARD	VB
VBFA [1]	–	–	heteroscedatic	ARD	VB
PEER [22]	–	–	hetroscedatic	ARD	VB
f-scLVM	spike-and-slab, sparse and dense factors	yes	heteroscedatic/nonGaussian	ARD	VB
Seeger et al. [20]	–	–	homoscedatic/non-Gaussian	None	VB
ZiFA [17]	–	–	heteroscedatic w/ zero inflation	None	EM
NSFA [12]	–	–	hetroscedatic	IBP	VB
Gao et al. [8]	TBP, sparse and dense factors	–	hetroscedatic	TBP	EM
Biswas et al. [19]	spike-and-slab	yes	heteroscedatic	None	MCMC

sparsity prior in conjunction with an ARD prior to infer which annotated factors are most relevant. Factor analysis with prior information has been utilized in early methods to reconstruct gene regulatory networks [19] Additionally, f-scLVM models the annotation as observed data that scales with the size of the expression dataset (Section 1.1.1), rather than using it to define a regulatory prior. This approach yields additional robustness across a wide range of different expression datasets (Fig. M3).

A third key aspect of factor analysis models is the noise model employed, ranging from simple homoscedastic Gaussian noise models [18, 22] to more complex approaches for modeling non-Gaussian noise, i.e. to account for over-dispersion [17]. To the best of our knowledge there is currently no existing method that combines non-Gaussian likelihood models and sparse factor analysis models. f-scLVM provides flexible likelihood models either modeling the observed data on a log Gaussian scale, as Poisson counts or using a Hurdle model.

Finally, factor analysis models use different inference schemes to fit model parameters. Many approaches employ accurate but slow MCMC methods, which tend to scale poorly to larger datasets. f-scLVM employs approximate Bayesian inference to achieve linear runtime complexity, thereby enabling its application to large datasets. For a tabular comparison of alternative methods, see Table 1.

3 Practical considerations, parameters and implementation

Variational Bayesian inference can be sensitive to implementation details such as parameter initialization and the update order. In the following we describe the specific strategy we use in f-scLVM .

3.1 Preprocessing

In the experiments we consider normalized single-cell RNA-seq dataset, following the primary analysis used in the respective source publication; see also Online Methods. When applying f-scLVM with the standard Gaussian noise model, we fit it on raw log count values ($log(count + 1)$) using mean centred expression values (per gene). When applying f-scLVM with the dropout noise-model, we employed the identical strategy, however without mean centring the data, such that observations with zero counts retain their value. When using f-scLVM in conjunction with the Poisson noise model, no log transformation was performed and the model is applied to the raw count values.

For each dataset, we reduced gene set annotations and only considered terms with at least 20 (expressed) assigned genes (15 for the more carefully curated MSigDB). Additionally, we reduced the set of genes and considered only expressed genes that were annotated to at least one pathway term.

3.2 Model initialisation, VB update schedule and convergence

Initialization of variational parameters The variational parameters of the regulatory weights $\tilde{\mathbf{W}}$ are initialized randomly by sampling from a unit variance normal distribution, scaled by $\frac{1}{\sqrt{K}}$, with K being the number of factors. The variational parameters of latent factors (columns of $\mathbf{X}$) that correspond to annotated factors are initialized using the first principal component calculated on the prior gene set of the corresponding factor. Dense *unannotated* factors without pathway information are initialized randomly by sampling from the prior (unit variance normal). Sparse unannotated factors are initialised using the first principal component of 20 randomly chosen highly variable genes (sampled from the top 100 most variable genes, sorted by variance).

The variational parameters of the regulatory sparsity prior $\gamma_{g,k}$ are initialized with the prior (Bernoulli prior with success probability $[\rho_{g,k}]$); for sparse unannotated factors, we initialise $\gamma_{g,k}$ corresponding to the 20 randomly chosen genes to 0.9. When a non-Gaussian noise model (dropout or Poisson noise model) is used, the pseudo-observations ($\tilde{\mathbf{Y}}$) are initialized using the observed data $\mathbf{Y}$.

Parameter update schedule The variational schedule updates $Q(\tilde{\mathbf{W}}, \mathbf{Z})$ first, followed by $Q(\boldsymbol{\alpha})$,$Q(\mathbf{X})$ and finally $Q(\boldsymbol{\tau})$. For the non-Gaussian noise models an additional update step for the pseudo observations $\tilde{\mathbf{Y}}$ is included. As the individual factors $\mathbf{X}_{:,k}$ should capture variation due to a particular biological process, it is important to minimize the risk of label switching, whereby the factor states do not match the regulatory annotation (see also [10]). This problem is specific to sparse factor models that incorporate prior information, where unlike standard FA the order of the factors is meaningful. To mitigate possible biases, we update the Q distributions of individual factors $Q_{:,k}$ in a randomized order, using different permutations in each model iteration. While this approach reduced ordering effects, we observed that the final solution is still affected by the update order of individual factors in the first iteration. To address this, we used a heuristic to determine the initial update order rather than random permutations for the first update cycle. This initial order is determined using a pre-training approach, for which we consider 50 update iterations: factors are ordered in increasing and decreasing order to correlation with

the first principal component on all annotated genes and the consensus order after 50 updates is used as initial permutation for updating f-scLVM . Empirically, we observed that this heuristics leads to improved convergence and more accurate estimates of the final factor relevance (Fig. M2).

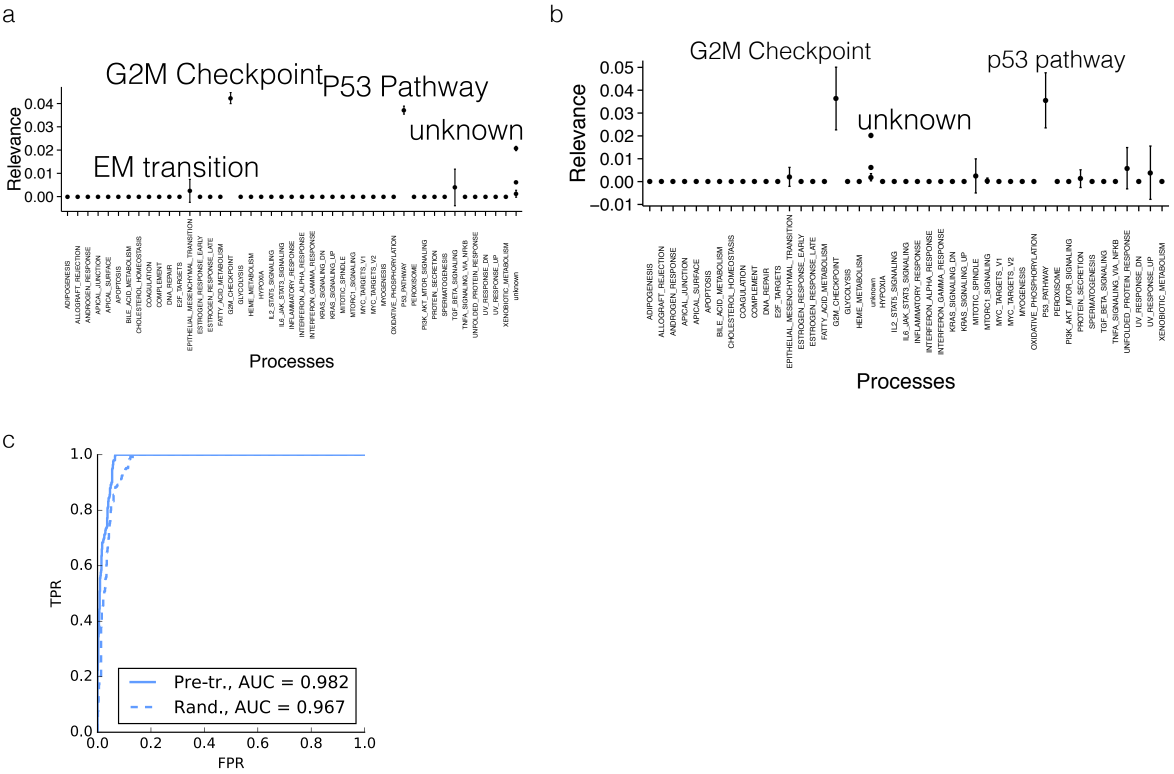

Supp. Meth. Figure M2: **Impact of pretraining to determine an initial factor update order. a,b)** Comparison of the inferred factor relevance for the cell-cycle staged mESC dataset (see also Supp. Fig. 4), using a bootstrap approach to assess robustness of the factor relevance. Results with pre-training are shown in **(a)**, analogous results without pre-training in **(b)**. In general, pre-training resulted in reduced variability across boot strap repeats, but with overall consistent interpretation. **c)** Comparison of the accuracy of f-scLVM on simulated data with default parameter settings (see Supplementary Table 1), either with or without (Rand.) pre-training (analogous to the results reported in main paper Fig. 2b). The pre-training approach resulted in slightly improved accuracy.

Monitoring convergence Model updates are performed until convergence, which was monitored using the reconstruction error. Alternatively, it is also possible to monitor the variational lower bound of the

marginal likelihood (Eq. (12)). However, this approach would increase the computational cost as an explicit evaluation of the bound almost doubles the per-iteration compute cost. In practice, we observed that monitoring the reconstruction error is sufficient. We considered up to 2,000 iterations of variational updates or until convergence of the reconstruction error ($\epsilon < 10^{-6}$ for 50 consecutive iterations) was achieved, whatever occurred first.

3.3 Learning unannotated factors

Similarly, f-scLVM has the ability to learn sparse unannotated factors, which can, for example, capture variation between cell types, that are not readily reflected in pathway annotations provided to the model. We model these factor by setting π to 0.1, reflecting our prior belief that roughly 10% of genes should be active in a sparse hidden factor. To facilitate efficient learning, we initialize our model by seeding each factor with 20 highly variable genes for which we set π to 0.99.

3.4 Applying f-scLVM to very large datasets

We implemented a series of additional measures to improve the practical performance and convergence rate of f-scLVM. Fist, our software implementation makes use of parallel processing capacities, if executed using a modern Python interpreter. Second, to facilitate inference on datasets with 10,000 cells or more, as well as when using larger numbers of pathway factors (e.g. > 200 in the REACTOME database), we provide support for a pre-scoring heuristic to reduce the number of factors that need to be fitted jointly. Specifically, we first fit factors independently using SVD on the prior annotated gene sets per factor. We then retain the 50 terms for which the first eigenvector explains most of the observed variance. While this approach is likely to overestimate the importance of individual factors (cf. Supp. Fig. 1), is effective for pruning pathways that are highly unlikely to be relevant. Third, the model allows deactivation of individual factors once they have extremely low relevance (using $\alpha_k/var(\mathbf{x}_k) > 10^{10}$ as a criterion). This early stopping approach is motivated by the observation that factors, once deactivated by the ARD prior, are unlikely to be reactivated in later stages of training.

3.5 Hyperparameter settings

In the experiments, we considered the following hyperparameters. For the spike-and-slab prior for annotated factors we choose an uninformative prior of $\pi = 0.5$. To model the annotation we set $1 - FPR$ to 99% and FNR to 0.1%, reflecting the belief that annotations are specific but include genes that are not necessary relevant in a given study. For the Gamma prior on α_k and τ_g, we chose the hyperparameters $a = 10^{-3}$ and $b = 10^{-3}$, which correspond to uninformative prior settings.

Scaling the gene set annotations with gene set size An additional parameter is n_{eff}, which corresponds to the effective number of cells based on which the annotation size is scaled to larger datasets. Technically, the likelihood term for the annotations $P(\mathbf{I}\,|\,\mathbf{Z})$ (Eq. (8)) is scaled by N/n_{eff}. This approach is equivalent to modeling a full set of gene set annotations for each n_{eff} cells in the dataset. In the experiments, we use n_{eff}, which means that the FNR and FPR settings for the prior annotations are relative to a dataset with 200 cells. Empirically, we confirmed the expected effect of scaling the annotation likelihood with the data likelihood (see also Sec. 1.1.1). When using a fixed annotation prior, the number of false positive

augmentations of gene sets (using the posterior on $\mathbf{Z}$) scaled approximately linear with the dataset size, which reflects that the inferred factors are increasingly decoupled from the annotation. In contrast, the likelihood scaling yielded robust and accurate results across a wider range of datast sizes (Fig. M3).

Determining the number of unannotated factors The number of unannotated factors to include in the model is in principle a hyperparameter that needs to be set by the user. First, for unannotated dense factors, we observe that the model is insensitive to the total number of such factors included. This is because the ARD prior is able to deactivate those factors that are not needed, leading to results that are robust across a larger range (see also Supp. Fig. 3h). In the experiments, we include 3 unannotated dense factors throughout.

The number of unannotated sparse factors requires further considerations, however. Because these factors are sparse and there exists no prior annotation to constrain the gene sets they effect, we find that the ARD prior less effectively regularizes their relevance. Consequently, sparse unannotated factors should only be activated if they are needed to explain the variation in the data. Empirically, we observed that if larger numbers of genes are activated in the annotated factors (more than 100%, see Sec. 3.6), this indicates that the annotation is not able to appropriately capture the variation in the data. This situation applied to the Zeisel data set as well as the human preimplentation Embroys (Supp. analyses Fig. SN1). To address this we fit 5 sparse hidden factors for these two datasets. Sparse hidden factors tend to capture variation between cell types, which are typically not well captured by annotated factors (Supp. Fig. 6).

3.6 Downstream analyses

The trained f-scLVM model can be used for different downstream analyses.

Factor relevance First, the posterior mean of the ARD score (factor-wise precision) $\hat{\alpha_k}$ is used as a measure for the relevance of an individual factor to drive expression variability. The inverse of this ARD score can be interpreted as the expected explained variance of the factor for the subset of genes with a regulatory effect. Larger values of $1/\hat{\alpha_k}$, which correspond to the expected variance explained by factor k, indicate larger relevance of factor k. When analysing the drivers of variability for selected subsets of cells only, the factor relevance can be mapped onto this subset without the need to recompute the model. This is achieved by re-weighting $1/\hat{\alpha_k}$ with the relative variance of the corresponding factor k within the subset of cells under consideration. To exclude factors that may be driven by outlying cells, we filtered the reconstructed factors based on the mean absolute deviation (mad) and excluded factors with mad less than .4 before calculating the relevance score. For the retina and Zeisel datasets, no such filtering was applied, due to the known presence of very small cell populations.

Visualization Second, the posterior distribution over annotated and unannotated factors $\mathbf{X}_{:,k}$ can be used to visualize cell states.

Gene set refinement By comparing the posterior distribution over the gene assignment to factors $z_{g,k}$ to the prior annotation $I_{g,k}$, it is possible to identify individual genes that were added to or removed from a given pathway factor k. In practice, we consider the posterior threshold .5 for annotating genes to factors.

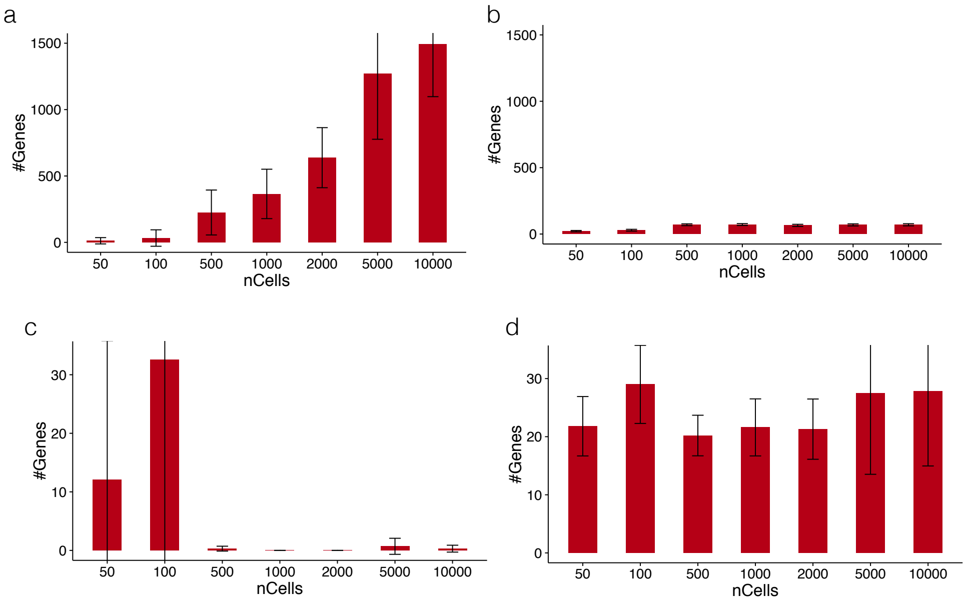

Supp. Meth. Figure M3: **Impact of the scaling of the annotation likelihood by the number of cells in the data.** Shown are results for different models using simulated pathway corruptions as shown in Supp. Fig. 4, considering increasing dataset sizes (cell count). **(a,b)** Number of false positive **(a)** and true positive **(b)** augmentations to pathway annotations without rescaling. **(c,d)** Analogous results when using the annotation likelihood rescaling described in Sec. 1.1.1. Without scaling the annotation likelihood, the inferred factors become decoupled for large dataset, which results in large numbers of false positive augmentations to the annotation (**(a)**). In contrast, the scaled annotation likelihood retains its relevance across datasets of different size, yielding decreased false positive **(c)** and increased true positive **(d)** augmentations for larger datasets.

Model residuals Finally, the inferred annotated and unannotated factors can also be used to estimate residual dataset. Residual data adjusted for the effect of a given factor k are derived using $\mathbf{Y}_{\text{residual}} = \mathbf{Y} - \mathbf{X}_{:,k}\mathbf{W}_k^{\text{T}}$ When the model was trained using the dropout noise model, the residuals were calculated using the pseudo-counts $\tilde{\mathbf{Y}}$. This approach performs an implicit imputation of zero count values.

References

[1] Hagai Attias. Inferring parameters and structure of latent variable models by variational bayes. In *Proceedings of the Fifteenth conference on Uncertainty in artificial intelligence*, pages 21–30. Morgan Kaufmann Publishers Inc., 1999.

[2] Hagai Attias. A variational bayesian framework for graphical models. *Advances in neural information processing systems*, 12(1-2):209–215, 2000.

[3] Matthew James Beal. *Variational algorithms for approximate Bayesian inference.* University of London, 2003.

[4] Christopher M Bishop. Bayesian pca. *Advances in neural information processing systems*, pages 382–388, 1999.

[5] Christopher M Bishop. Pattern recognition. *Machine Learning*, 2006.

[6] Barbara E Engelhardt and Matthew Stephens. Analysis of population structure: a unifying framework and novel methods based on sparse factor analysis. *PLoS Genet*, 6(9):e1001117, 2010.

[7] Greg Finak, Andrew McDavid, Masanao Yajima, Jingyuan Deng, Vivian Gersuk, Alex K Shalek, Chloe K Slichter, Hannah W Miller, M Juliana McElrath, Martin Prlic, et al. Mast: a flexible statistical framework for assessing transcriptional changes and characterizing heterogeneity in single-cell rna sequencing data. *Genome biology*, 16(1):1–13, 2015.

[8] Chuan Gao and Barbara E Engelhardt. A sparse factor analysis model for high dimensional latent spaces. In *NIPS: Workshop on Analysis Operator Learning vs. Dictionary Learning: Fraternal Twins in Sparse Modeling*, 2012.

[9] Zoubin Ghahramani, Matthew J Beal, et al. Variational inference for bayesian mixtures of factor analysers. In *NIPS*, volume 12, pages 449–455, 1999.

[10] Ajay Jasra, CC Holmes, and DA Stephens. Markov chain monte carlo methods and the label switching problem in bayesian mixture modeling. *Statistical Science*, pages 50–67, 2005.

[11] Michael I Jordan, Zoubin Ghahramani, Tommi S Jaakkola, and Lawrence K Saul. An introduction to variational methods for graphical models. *Machine learning*, 37(2):183–233, 1999.

[12] David Knowles and Zoubin Ghahramani. Nonparametric bayesian sparse factor models with application to gene expression modeling. *The Annals of Applied Statistics*, pages 1534–1552, 2011.

[13] Miguel Lázaro-gredilla and Michalis K Titsias. Spike and slab variational inference for multi-task and multiple kernel learning. In *Advances in neural information processing systems*, pages 2339–2347, 2011.

[14] Jeffrey T Leek and John D Storey. Capturing heterogeneity in gene expression studies by surrogate variable analysis. *PLoS Genetics*, 3(9):e161, 2007.

[15] David JC MacKay. Bayesian nonlinear modeling for the prediction competition. *ASHRAE transactions*, 100(2):1053–1062, 1994.

[16] Leopold Parts, Oliver Stegle, John Winn, and Richard Durbin. Joint genetic analysis of gene expression data with inferred cellular phenotypes. *PLoS Genet*, 7(1):e1001276, 2011.

[17] Emma Pierson and Christopher Yau. Zifa: Dimensionality reduction for zero-inflated single-cell gene expression analysis. *Genome Biol*, 16:241, Nov 2015.

[18] Sam Roweis and Zoubin Ghahramani. A unifying review of linear gaussian models. *Neural computation*, 11(2):305–345, 1999.

[19] Chiara Sabatti and Gareth M James. Bayesian sparse hidden components analysis for transcription regulation networks. *Bioinformatics*, 22(6):739–746, 2006.

[20] Matthias Seeger and Guillaume Bouchard. Fast variational bayesian inference for non-conjugate matrix factorization models. In *Proceedings of the 15th international conference on artificial intelligence and statistics*, number EPFL-CONF-174931, 2012.

[21] O Stegle, K Sharp, J Winn, and M Rattray. A comparison of inference in sparse factor analysis models. Technical report, Technical report, 2010.

[22] Oliver Stegle, Leopold Parts, Richard Durbin, and John Winn. A bayesian framework to account for complex non-genetic factors in gene expression levels greatly increases power in eqtl studies. *PLoS computational biology*, 6(5):e1000770, 2010.

[23] Michael E Tipping and Christopher M Bishop. Probabilistic principal component analysis. *Journal of the Royal Statistical Society: Series B (Statistical Methodology)*, 61(3):611–622, 1999.

[24] Ryo Yoshida and Mike West. Bayesian learning in sparse graphical factor models via variational mean-field annealing. *The Journal of Machine Learning Research*, 11:1771–1798, 2010.

[25] Mingyuan Zhou, Haojun Chen, Lu Ren, Guillermo Sapiro, Lawrence Carin, and John W Paisley. Non-parametric bayesian dictionary learning for sparse image representations. In *Advances in neural information processing systems*, pages 2295–2303, 2009.
