# Supplementary material for: f-scLVM: scalable and versatile factor analysis for single-cell RNA-seq
Source: Genome Biol. 2017 Nov 7;18:212. doi: 10.1186/s13059-017-1334-8 (PMC5674756; doi:10.1186/s13059-017-1334-8)
Supplement: Supplementary file 2 — Supplementary figures and tables. (PDF 14372 kb) [file 13059_2017_1334_MOESM2_ESM.pdf]

Supplementary material

SUPPLEMENTARY TABLES 2

SUPPLEMENTARY FIGURES 3

SUPPLEMENTARY ANALYSES 15

HUMAN PREIMPLANTATION EMBRYOS 15
PARALLEL DNA-METHYLATION AND TRANSCRIPTOME PROFILED MOUSE ES CELLS 17
T-CELLS 19

Supplementary Tables

Supplementary Table 1 | Parameter values for simulated datasets. Datasets were simulated considering varying degrees of overlap between factors, increasing the numbers of simulated annotated- and unannotated (confounding) factors, different false negative/false positive rates for the simulated annotation (FNR/FPR), simulated swapped assignments of genes to factors, increasing numbers of simulated cells, gene sets of different sizes, and alternative parameter values for simulating dropout effects. The default setting for each parameter is highlighted in bold. Parameter were varied one at a time with the other parameters held at the default value. Left: Simulation parameters for standard log Gaussian noise. Right: Parameter settings when dropout noise was simulated.

Gene set overlap	Annotated factors	Unannotated factors	FN [%]	FP [%]	Gene swap [%]	Cell count	Gene set size	Expression quantile	Drop (λ)
0	2	0	1	**1**	**0**	50	20-50	0.01	0.4
0.1	3	**1**	**5**	2	1	**100**	50-100	0.02	0.6
**0.3**	4	2	10	3	5	200	100-200	**0.04**	0.8
0.5	**5**	3	15	5	10	500	**20-950**	0.1	**1.0**
0.7	7	4	25	10	25			0.5	1.2
	9	7	50						1.5

Supplementary table 2 | GO enrichment for differentially expressed genes between the astrocyte sub populations. Shown are the 20 most significantly enriched GO terms (using the R package topGO and the elim algorithm) based on the set of 1,024 significant differentially expressed genes between the astrocyte populations (FDR<10%).

	GO.ID	Term	Significant	p-value
1	GO:0006954	inflammatory response	28/39	0.0049
2	GO:0090092	regulation of transmembrane receptor pro...	16/21	0.0138
3	GO:0031334	positive regulation of protein complex a...	14/18	0.0161
4	GO:0030509	BMP signaling pathway	12/15	0.0183
5	GO:0071772	response to BMP	12/15	0.0183
6	GO:0071773	cellular response to BMP stimulus	12/15	0.0183
7	GO:0032273	positive regulation of protein polymeriz...	10/12	0.0200
8	GO:0030510	regulation of BMP signaling pathway	10/12	0.0200
9	GO:0018107	peptidyl-threonine phosphorylation	8/9	0.0202
10	GO:0018210	peptidyl-threonine modification	8/9	0.0202
11	GO:0030514	negative regulation of BMP signaling pat...	8/9	0.0202
12	GO:0001837	epithelial to mesenchymal transition	8/9	0.0202
13	GO:0007498	mesoderm development	8/9	0.0202
14	GO:0007009	plasma membrane organization	15/20	0.0215
15	GO:0009952	anterior/posterior pattern specification	13/17	0.0255
16	GO:0032924	activin receptor signaling pathway	5/5	0.0321
17	GO:0006368	transcription elongation from RNA polyme...	5/5	0.0321
18	GO:0006970	response to osmotic stress	5/5	0.0321
19	GO:0019882	antigen processing and presentation	5/5	0.0321
20	GO:0050766	positive regulation of phagocytosis	5/5	0.0321

Supplementary Figures

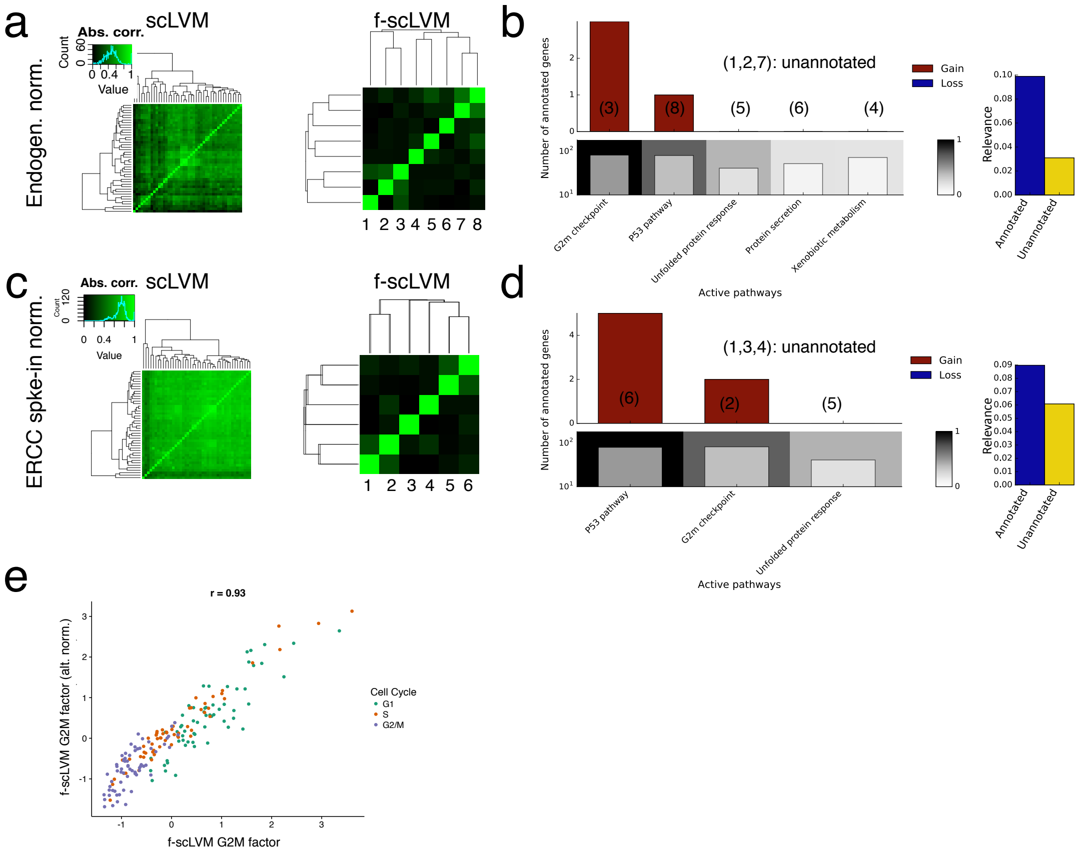

Supplementary Figure 1 | Analysis for the set of 182 cell-cycle staged mouse embryonic stem cells, considering alternative normalization strategies and different methods for inferring biological drivers of gene expression variability. (a) Comparison of independent and joint factor inference on mouse embryonic stem cells (mESC) staged for the cell cycle. Shown are pair-wise correlation coefficients of inferred factors, either considering scLVM independently applied to each of 44 gene sets derived from the core molecular signature database (MSIGDB; left) or when considering the subset of eight active factors identified by f-scLVM (right). While the factors identified by the independent model were correlated (average |r|=0.45), f-scLVM retrieved largely uncorrelated components, suggesting these factors tag distinct biolgoical processes (average |r|=0.09). **(b)** Factor relevance and gene set augmentation results from f-scLVM. Bottom pannel: size and relevance of the 5 annotated factors identifeid by the model. Top pannel: number of genes added to individual factors by the model. Right panel: cumulative relevance of annotated and unannotated factors. **(c-d)** Results analogous to those shown in **a,b,** however when considering a normalization strategy based on size factors calculated using ERCC spike-ins, which retains absolute variation in gene expression levels between cells. **(e)** Scatterplot of the G2M cell cycle factor, comparing the factor inference of f-scLVM when applied to data normalized using either of the two strategies. f-scLVM consistently recovered the main drivers of gene expression heterogeneity (G2M checkpoint, P53 pathway), irrespective of the choice of data normalization **(b,d,e)**.

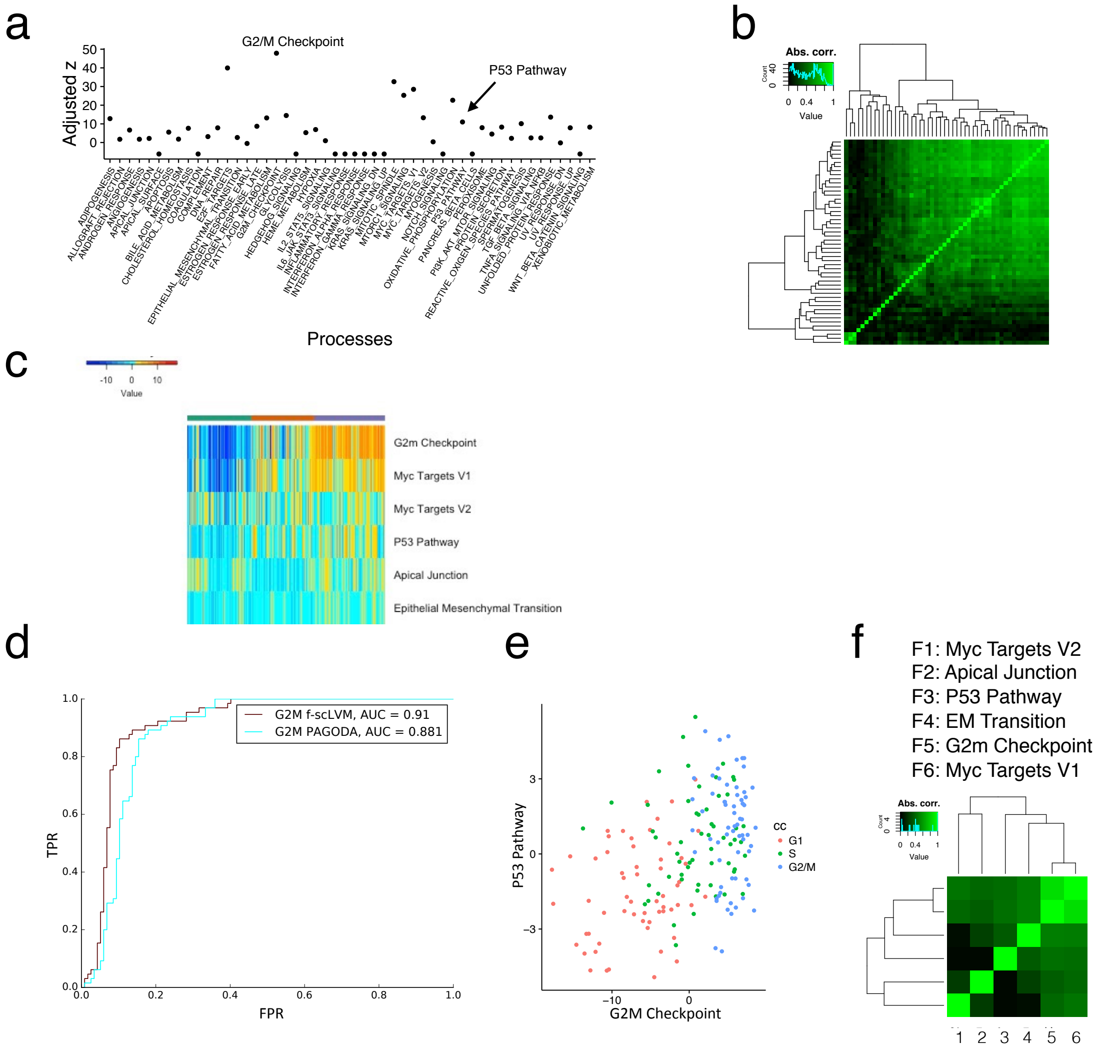

Supplementary Figure 2 | Additional analyses for the set of 182 cell-cycle staged mouse embryonic stem cells using PAGODA. (a) Raw factor relevance determined using the weighted PCA approach in PAGAODA, identifying a large number of putatively active pathways. **(b)** Pairwise correlation analysis of the inferred factors, revealing strong correlations similar to the SVD-based scLVM model (cf. Supp. Fig. 1a). **(c)** Reduced set of factors following PAGODA post-processing steps, resulting in clusters of factors that include G2M Checkpoint and the P53 pathway as main components. **(d)** Predictive accuracy to classify true G2/M cells, using either the G2M checkpoint factor inferred when using PAGODA (cyan) or f-scLVM (red). The f-scLVM factor more accurately discriminates the cells into two populations. **(e,f)** Correlations of factors inferred using PAGODA, **(e)** bivariate visualization using the G2M checkpoint and P53 factor; **(f)** pairwise correlation of the factors inferred when using PAGOA. In comparison to f-scLVM (cf. Supp. Fig. 1a,c), PAGODA yielded factors with a clear covariance structure, which is mainly because individual factors are inferred independently in this model. In contrast, f-scLVM performs joint inference of annotated and unannotated factors, yielding a smaller number of factors that capture independent components of variation.

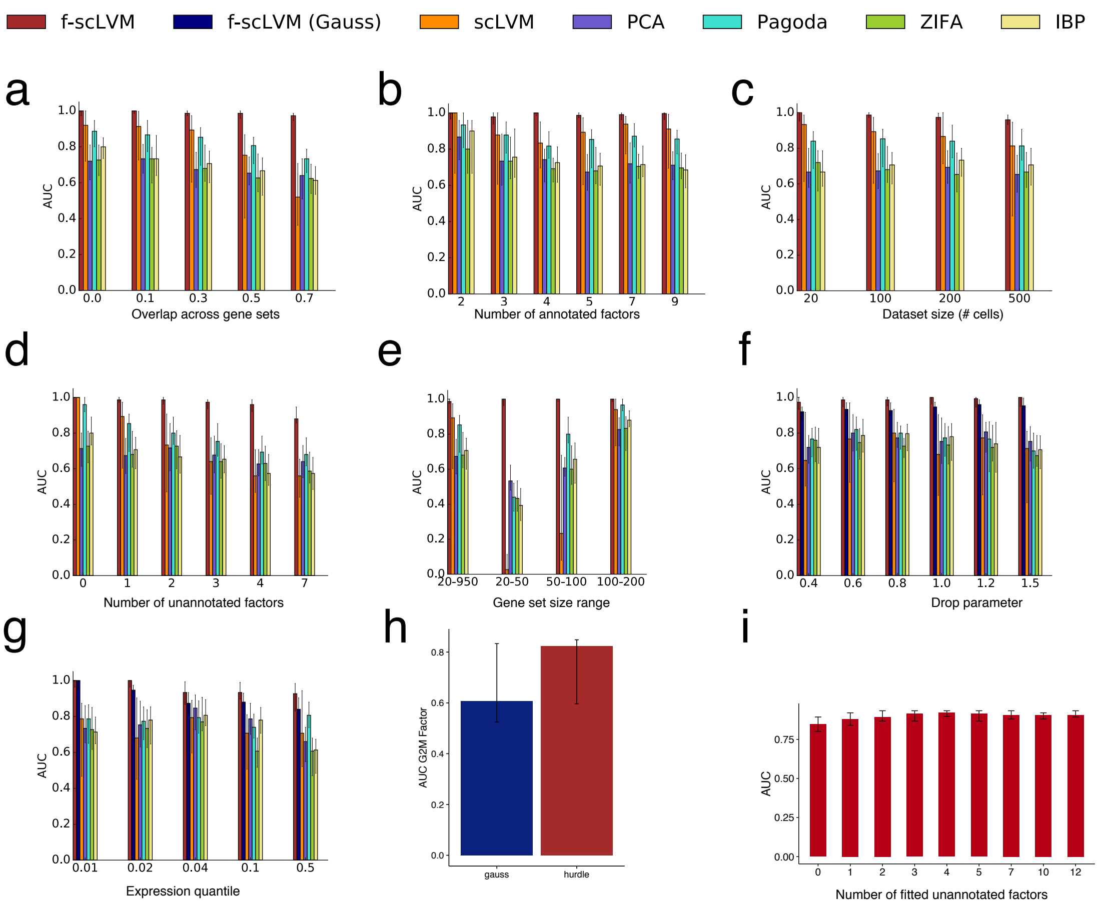

Supplementary Figure 3 | Additional results using simulated data. (a-e) Performance of f-scLVM and alternative methods for recovering true simulated drivers of gene expression heterogeneity, assessed using the area under the receiver operating characteristics (ROC). Results are for varying simulation parameters: **(a)** increasing gene set overlap between simulated pathway factors**, (b)** increasing numbers of simulated annotated factors**, (c)** increasing dataset sizes (number of cells), **(d)** varying numbers of simulated dense unannotated (confounding) factors and **(e)** considering gene sets of different size. **(f,g)** Performance of f-scLVM and alternative methods when simulating dropout effects, which are typical for sparse sequencing datasets (see Supp. Table 1). Shown are results for the same models as considered in **(a-e)**, and additionally a variant of scLVM with a Gaussian likelihood model that does not account for dropout (f-scLVM-Gauss). **(f)** Results for increasing lower quantiles of the expression distribution for which dropout effects are simulated. **(g)** Results for increasing values of the dropout rate parameter (λ), which determines the dependency of the dropout rate and the mean expression values. **(h)** Perfromance of f-scLVM to recover the true G2M state of a staged mESC when simulating drop-out effects (simulated droput of 50% quantile of expressed genes) for both likelihood models for all cells in the staged mESC dataset. As for the simulated data, f-scLVM outperforms f-scLVM Gauss. **(i)** Accuracy of f-scLVM for recovering true simulated drivers when fitting increasing numbers of unannotated factors in the model (default is 3), when two factors are simulated. The model accuracy saturated when at least two factors are fit, which confirms the robustness of the f-scLVM when fitting additional unannotated dense factors. Individual bars show aggregate results from 50

repeat experiments per setting, with their height corresponding to the median AUC and the error bars corresponding to 25% and 75% quantiles. In each simulation experiments all parameters except for the parameter under consideration were retained at their default values (see Supp. Table 1).

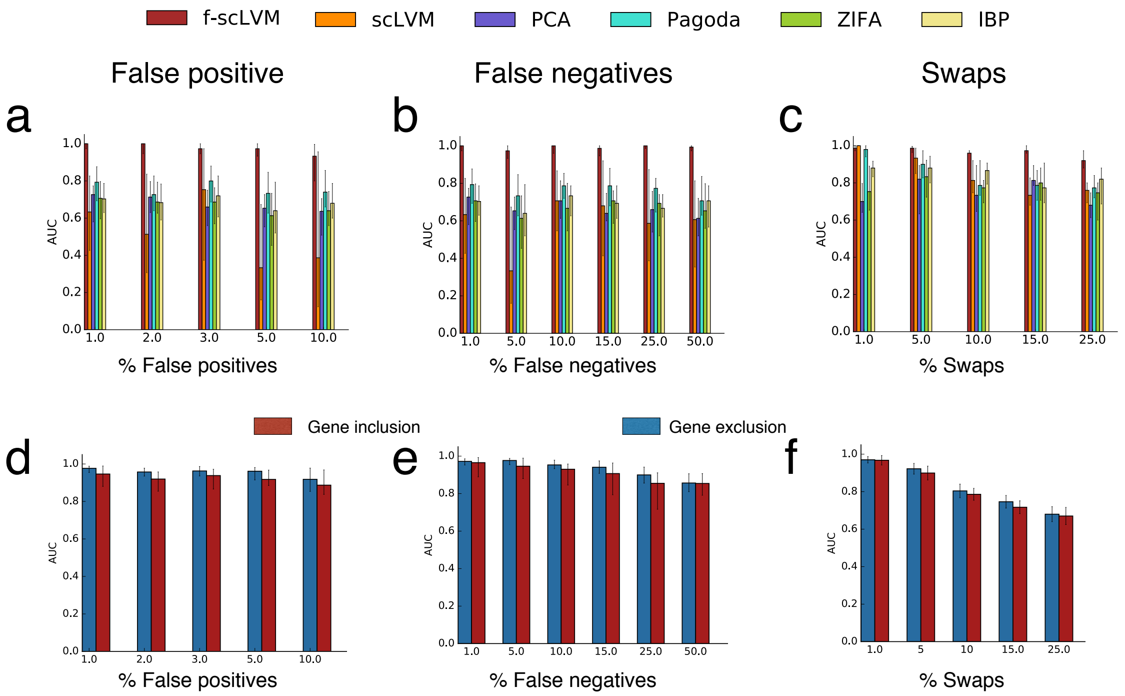

Supplementary Figure 4 | f-scLVM performance for simulated errors in the gene set annotation. (a-c) Area under the receiver operating characteristics (ROC), comparing the performance of f-scLVM and alternative methods for recovering true drivers of expression heterogeneity when introducing different types of errors in the annotation that is passed to the respective methods: **(a)** simulated false positive assignments of genes to gene sets, **(b)** simulated false negative assignments of genes to gene sets and **(c)** permuting increasing fractions of genes between gene sets of active factors. **(d-f)** Accuracy of f-scLVM for augmenting the provided gene set annotation for the corresponding simulations, considering the accuracy for including and excluding genes from the corrupted annotation, based on the model posterior distribution over the indicator variable that assigns genes to factors (Methods). Individual bars show aggregate results from 50 simulations with their height corresponding to the median AUC and the error bars corresponding to 25% and 75% quantiles. In each simulation experiments all parameters except for the parameter under consideration were retained at their default values (see Supp. Table 1).

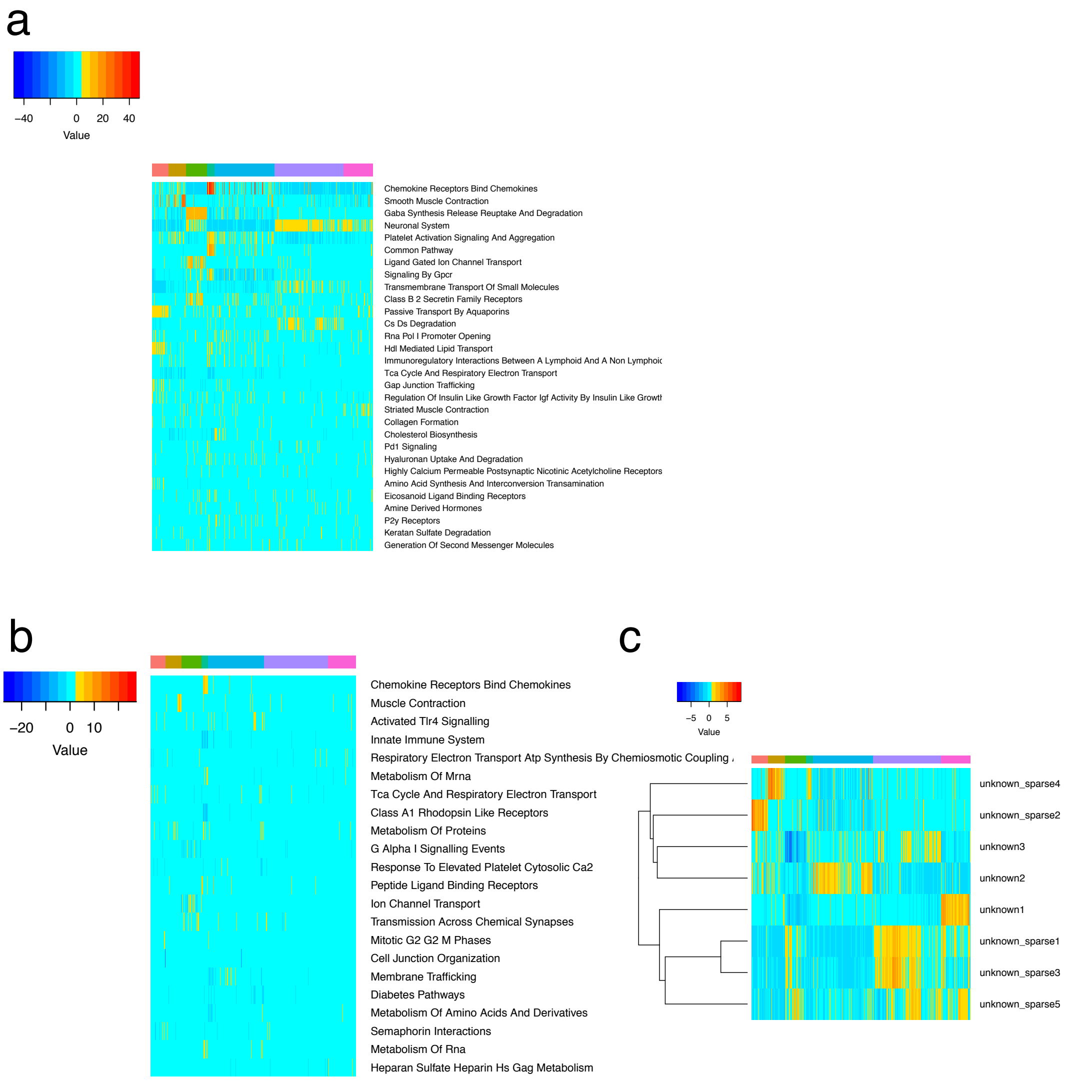

Supplementary Figure 5 | Additional analyses for the Zeisel dataset, including comparative results when using PAGODA. (a) Heatmap showing the top 30 processes identified by PAGODA after collapsing redundant factors (Methods). **(b)** Corresponding Heatmap for the top annotated factors identified by f-scLVM, which includes factors that were also identified by PAGODA, such as Chemokine Receptors Bind Chemokines, as well as factors outside the PAGODA set, including Innate immune system. **(c)** Aditional dense and sparse unannoatated factors identified by f-scLVM for the same dataset. While annotated factors predominantly resolved intra-cell type variation, unannotated factors tended to capture inter-cell type variation that cannot be readily captured by the annotated factors. Colors encode factor activity.

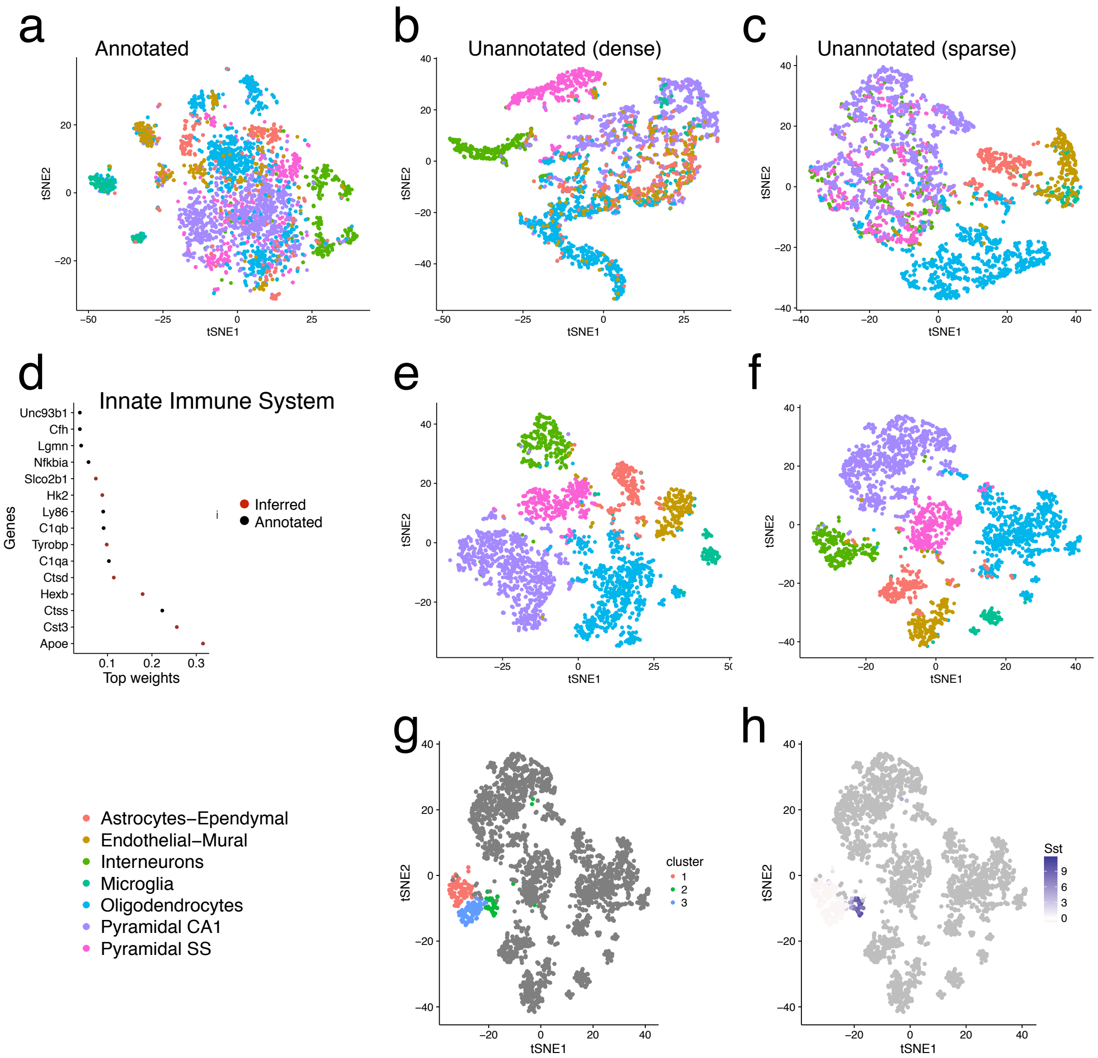

Supplementary Figure 6 | Additional analyses for the Zeisel dataset, including gene-set completion and cell state variation captured by different factors. (a-c) t-SNE visualization of the single-cell variation catpured by annotated factors **(a)**, as well as dense **(b)** and sparse **(c)** unannotated factors. While annotated factors predominantly resolved intra-cell type variation, unannotated factors tended to capture inter-cell type differences that cannot be readily captured by the annotated factors. **(d)** Relative weight of annotated and genes added by the model for the innate immune system factor. Several genes with a known implication in innate imunity were identified, including Apoe [1] and Hexb [2]. **(e-h)** Effect of adjusting for unwanted varaition by regressing out the most relevant dense unannotated factor. **(e)** t-SNE on unadjusted data. **(f)** t-SNE on adjusted data, revealing additional substructure in the neuronal cell population. The identified cell clusters **(g)** correspond to three well-characterized groups of neurons [3]. An ANOVA on gene expression using the cell clusters revealed 782 significant marker genes (FDR<10%), with Sst being the most significant gene. Sst is a canonical marker gene for the aforementioned subpopulations [3] **(h)**.

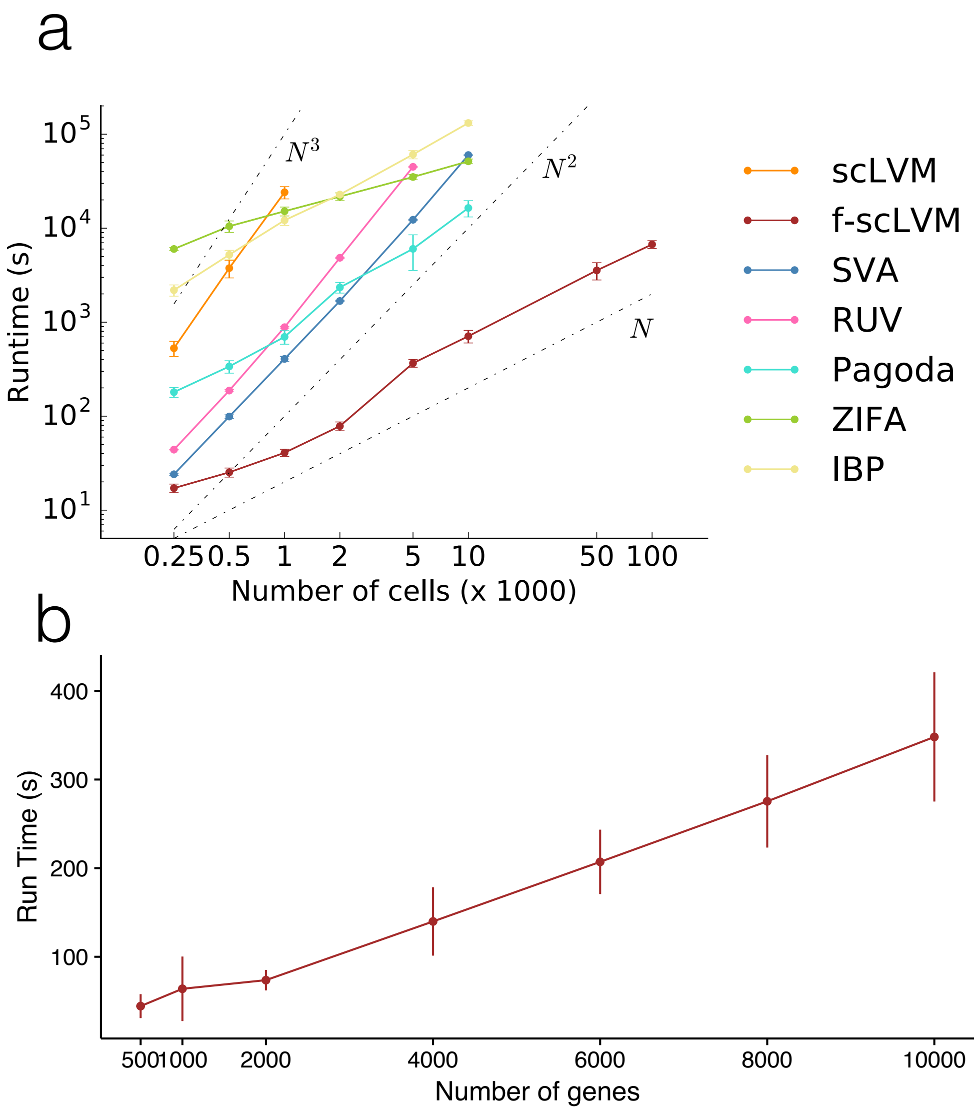

Supplementary Figure 7 | Additional results for the runtime comparisons. (a) Considering additional methods. Comparison of the empirical runtime when fitting f-scLVM and alternative methods based on factor analysis (RUV, SVA, scLVM, PAGODA, ZIFA, IBP). Considered are datasets with increasing numbers of cells and 6,000 genes. **(b)** Empirical runtime when fitting f-scLVM for an increasing number of genes and 100 cells. Shown are empirical runtimes obtained when fitting these models using 8 cores of an Intel(R) Xeon(R) CPU E5-2660 v3 @ 2.60GHz. None of the existing methods could be applied to the largest dataset.

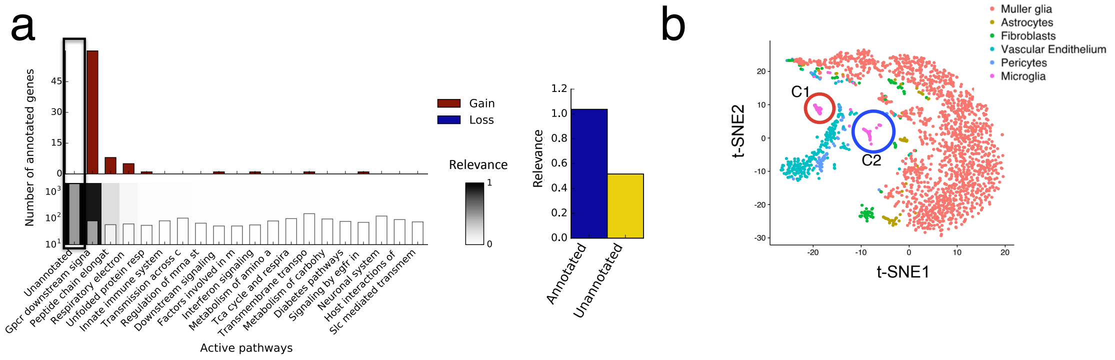

c

	GO.ID	Term	Significant	p-value
1	GO:0060249	anatomical structure homeostasis	27/30	0.0068
2	GO:0030217	T cell differentiation	22/24	0.0091
3	GO:0042113	B cell activation	16/17	0.0159
4	GO:0043903	regulation of symbiosis, encompassing mu...	15/16	0.0218
5	GO:0046631	alpha-beta T cell activation	10/10	0.0247
6	GO:0046632	alpha-beta T cell differentiation	10/10	0.0247
7	GO:0033077	T cell differentiation in thymus	10/10	0.0247
8	GO:0090100	positive regulation of transmembrane rec...	10/10	0.0247
9	GO:0071594	thymocyte aggregation	10/10	0.0247
10	GO:0030183	B cell differentiation	14/15	0.0299
11	GO:0048738	cardiac muscle tissue development	14/15	0.0299
12	GO:0050792	regulation of viral process	14/15	0.0299
13	GO:1903900	regulation of viral life cycle	14/15	0.0299
14	GO:0043412	macromolecule modification	219/297	0.0322
15	GO:0001819	positive regulation of cytokine producti...	21/24	0.0343

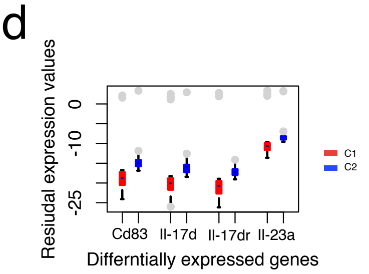

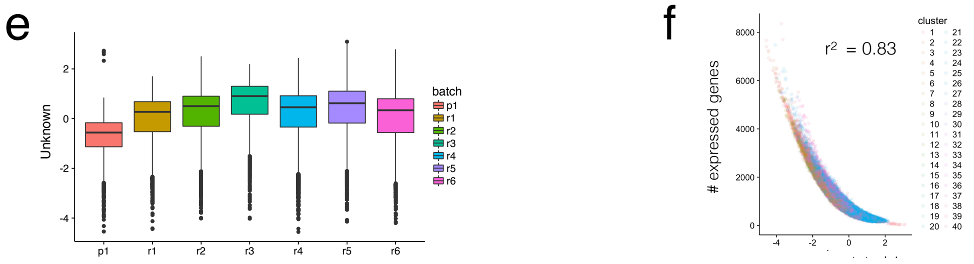

Supplementary Figure 8 | Additional results for the retina cells profiled using drop-seq. (a) Factor relevance, for the top 20 factors (annotated and unannotated factors) identified by f-scLVM for the subset of 2,145 cells considered in Fig. 5b,c and in (**b**). The most relevant factor was a dense unannotated factor, which was regressed out for estimating residual datasets ((**b)** and Fig. 5c). **(b)** Visualization of aforementioned subset of cells using non-linear t-SNE embedding applied to the residual dataset (see (**a**)). Colors correspond to the cell types identified in [4]. Red and blue circles annotate two subpopulations of microglia cells that could only be detected on the adjusted data (cf. Fig. 5c). **(c)** GO term enrichment for the set of 992 differentially expressed genes (FDR<1%) between the microglia subpopulations identified in **(b)**. **(d)** Several of the most differentially expressed genes play important roles in microglia activation, including Cd83 [5], and genes from the Il-6 family such as Il-17d, Il-17dr and Il-23a [6-8]. The discovered gene sets were implicated in processes related to activation of T-cells and B-cells, which are hallmarks of microglia activation, suggesting that one subpopulation consists of activated microglia. **(e,f)** Relationship between the most relevant unannotated factor (grey box in **a**) used to calculated residual datasets (cf. **(a)**) and experimental batch **(e)** and cellular detection rate **(f)**.

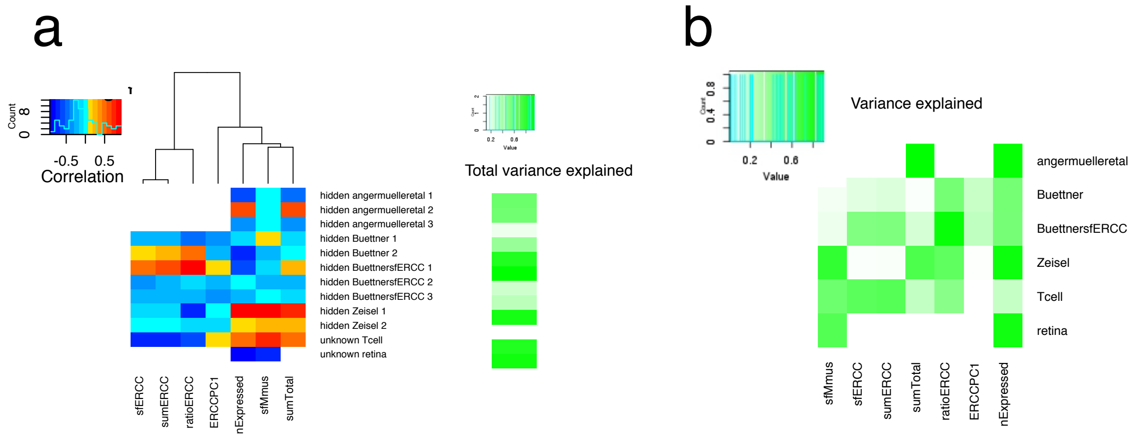

Supplementary Figure 9 | Associations between inferred unannotated dense factors and technical covariates. (a) Left: Correlation coefficients beween unannotaed dense factors inferred by f-scLVM and technical covariates across different scRNA-seq datasets. Right: Cumulative variance explained when considering all available technical covariates. Unannotated factors were frequently associated with covariates with known relevance for scRNA-seq, including the number of reads mapped to spike-ins (sumERCC), ERCC-derived size factor (sfERCC), PC1 derived from ERCC spike-ins (PC1ERCC), the number of expressed genes/cellular detection rate (nExpressed), the total number of mapped reads (sumTotal) and DE-seq size factor derived from reads mapped to endogenous genes (sfMmus). **(b)** Cumulative proportion of variance explained by all unannotated factors for individual technical covariates.

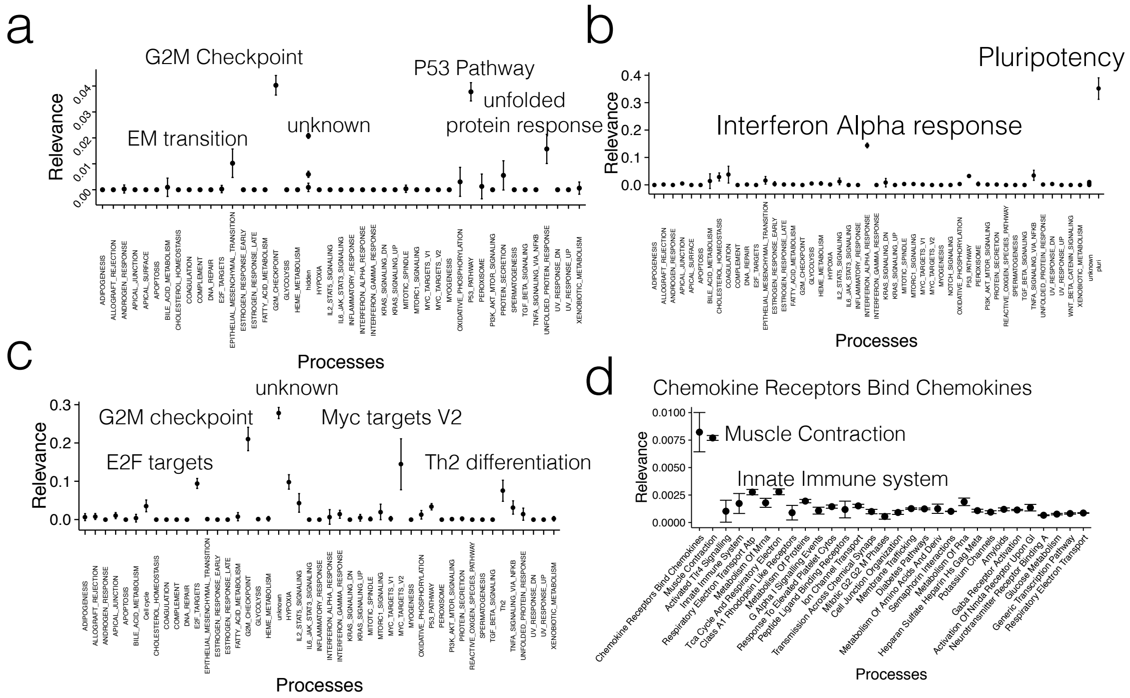

Supplementary Figure 10 | Robustness of f-scLVM factor relevance. Robustness was assessed using random sampling, repeatedly using a random subset of 80% of the cells to fit the model. Shown are the mean factor relevance and plus or minus one standard deviation confidence estimates derived from 10 sampling repetitions. Results for the cell-cycle staged mESC dataset **(a)**, the mESC dataset profiled using parallel DNA-methylation and transcriptome sequencing, **(b)** the T-cell dataset **(c)** and the Zeisel neuron data **(d)**.

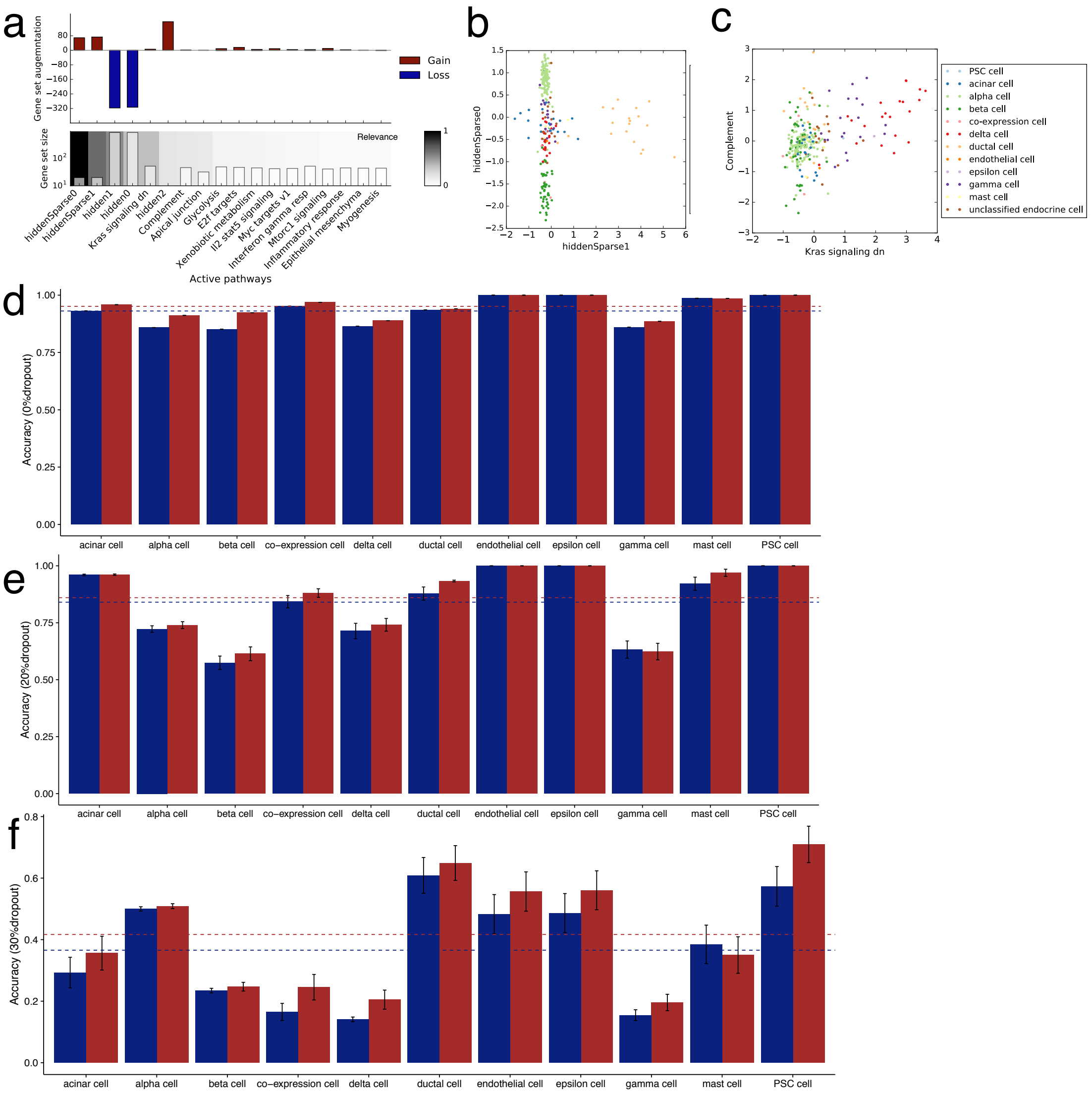

Supplementary Figure 11 | f-scLVM discrminates known cell types under noisy conditions. We assessed the ability of f-scLVM with Gaussian and hurdle noise model to discriminate known cell types within the human pancreas when simulating increasing rates of dropout (see also sections Supplementary analyses and Simulation Study (main text)). Factor relevance, for the most relevant factors (annotated and unannotated factors) identified by f-scLVM **(a).** Overall, the most relevant factors were sparse unannotated (hidden) factors that separated well the known cell types **(b).** The most relevant annotated factors were KRAS signaling and Complement System, with the Complement factor mainly separating ductal cells and the KRAS factor separating Delta and Gamma cells **(c).** The ability of f-scLVM to separate cell types based on sparse hidden factors degraded with increased dropout for both noise models; f-scLVM with dropout noise model consistently outperformed the Gaussian noise model with increased overall accuracies of up to 14% (for 30% dropout). Blue bars correspond to the Gaussian noise model, red bars to the hurdle noise model. Dashed lines represent the respective mean accuracy for Gaussian and hurdle noise model **(d-f).**

Supplementary analyses

Human preimplantation embryos

We applied f-scLVM to 1,529 human cells from 88 male and female embryos at different developmental stages ranging from E3 to E7. Consequently, the major drivers of expression variation are expected to be associated with developmental processes and sex-specific effects between embryos [9]. f-scLVM was fit using the MSigDB annotations, additional modelling unannotated sparse factors, which were required to fully capture variation outside the gene annotation (Methods). The most relevant annotated factors identified, such as TGF-Beta signaling or epithelia mesenchymal transition, primarily captured variation within developmental stages (Fig. SN1a-d). Interestingly, the factor oxidative phosphorylation accurately differentiated cells with different sexes (Fig. SN1a,d), which is consistent with documented differences in glucose and amino acid utilization between female and male preimplantation embryos [10]. Notably, 8 of the top 20 genes pre-annotated to this factor (largest weights, Fig. SN1e-f) were also identified as differentially expressed between sex in the primary analysis of the data, including NDUFA1 ($p = 5.2e\text{-}20$), SLC25A5 ($p = 2.6e\text{-}37$) and HSD17B10 ($p = 7.5e\text{-}20$). Additionally, f-scLVM identified 7 genes not in the annotation that were added to the gene set. These genes showed consistent changes between sex and were correlated to the pre-annotated genes. Several of these genes are known to be sex-linked genes such as XIST (Fig. SN1f).

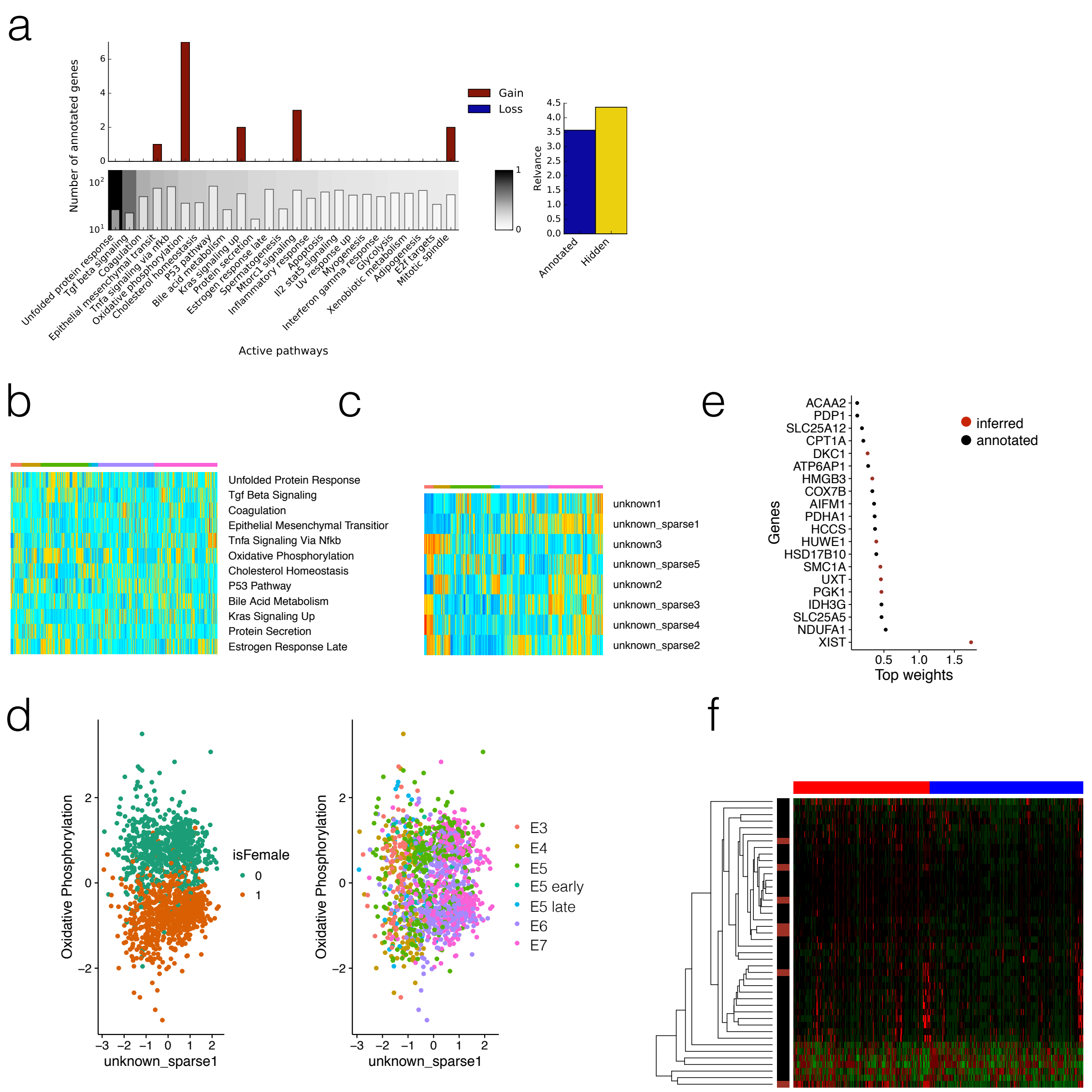

Figure SN1 | Application of f-scLVM to 1,529 cells from Human Preimplantation Embroys. (a) Relevance of individual factors as determined by f-scLVM based on the REACTOME annotation (left) and cumulative relevance of annotated and unannotated factors (right). **(b-c)** Heatmaps for most relevant annotated **(b)** and unannotated **(c)** factors identified by the model. The colour bar indicates developmental stages. Annotated factors tended to resolve intra-stage variation, whereas unannotated factors primarily captured inter-stage variation**. (d,e)** Bivariate visualization of cells using the inferred annotated factor oxidative phosphorylation and a sparse unannotated factor, considering cells labeled by sex **(d)** or developmental stage **(e)**. The Oxidative phosphorylation factor separates cells by sex while the unannotated sparse factor separates cells by developmental stage. **(e)** Relative weight of annotated and genes added by the model for the top 20 genes associated to the oxidative phosphorylation factor, including 7 genes added by f-scLVM. **(f)** Newly identified genes were correlated to pre-annotated genes, suggesting that the factor identity related to oxidative phosphorylation is maintained (columns correspond to cells ordered by sex: red = male, blue = female; rows correspond to members of the relevant gene set: black = pre-annotated, red = added by f-scLVM).

Parallel DNA-methylation and transcriptome profiled mouse ES cells

We applied f-scLVM to a set of 61 serum-cultured mESCs in G0/G1 phase profiled using parallel DNA methylation and transcriptome sequencing (scM&T-seq) [11]. We followed the preprocessing, QC and normalization described in the primary publication [11] and considered log-transformed normalized gene expression data of 61 cells for analysis using f-scLVM. Following the primary analysis, we included a set of 86 literature-curated pluripotency genes as an additional candidate gene set to augment the MSigDB annotation. The model-based factor relevance of f-scLVM identified this pluripotency factor as the most relevant diver of expression variation (Fig. SN2a). This is consistent with the expected cell-to-cell heterogeneity in pluripotency of serum-grown mESCs, manifesting in heterogeneous expression of key pluripotency marker genes [12, 13]. Next, we explored links between the inferred pluripotency factor and DNA methylation in the same set of cells. We observed a clear correlation between pluripotency and genome-wide methylation rate ($P<1e-5$, Fig. SN2b). This global effect is consistent with previous studies based on ensembles of cells, which reported an association between genome-wide methylation rate and pluripotency [14]. Additionally, we considered using the inferred pluripotency factor to test for associations with gene body methylation of individual genes genome-wide, which is conceptually similar to the approaches taken in epigenome-wide association analyses in population studies [15]. This revealed 8,124 genes where gene body methylation was significantly associated with pluripotency ($FDR < 10\%$, Fig. SN2c). For comparison, we also considered associations with the first PC on gene expression levels (3,907 associations; FDR 10%, Fig. SN2d) as well as a single-gene methylation-expression association as in [11] (43 associations; $FDR<10\%$, Fig. SN1e). These alternative strategies yielded markedly fewer significant associations. We also considered alternative genomic contexts (Fig. SN2g) and observed similar trends. The increase in power when using the inferred factor in association analyses shows that using latent variables is an effective approach for reducing noise in the experimental assay. This has clear advantages when analyzing single-cell transcriptome data, which are inherently prone to noise and technical sources of variation [16, 17]. At the same time, the inferred factors have a clear interpretation, unlike factors such as those derived using conventional SVD-based methods.

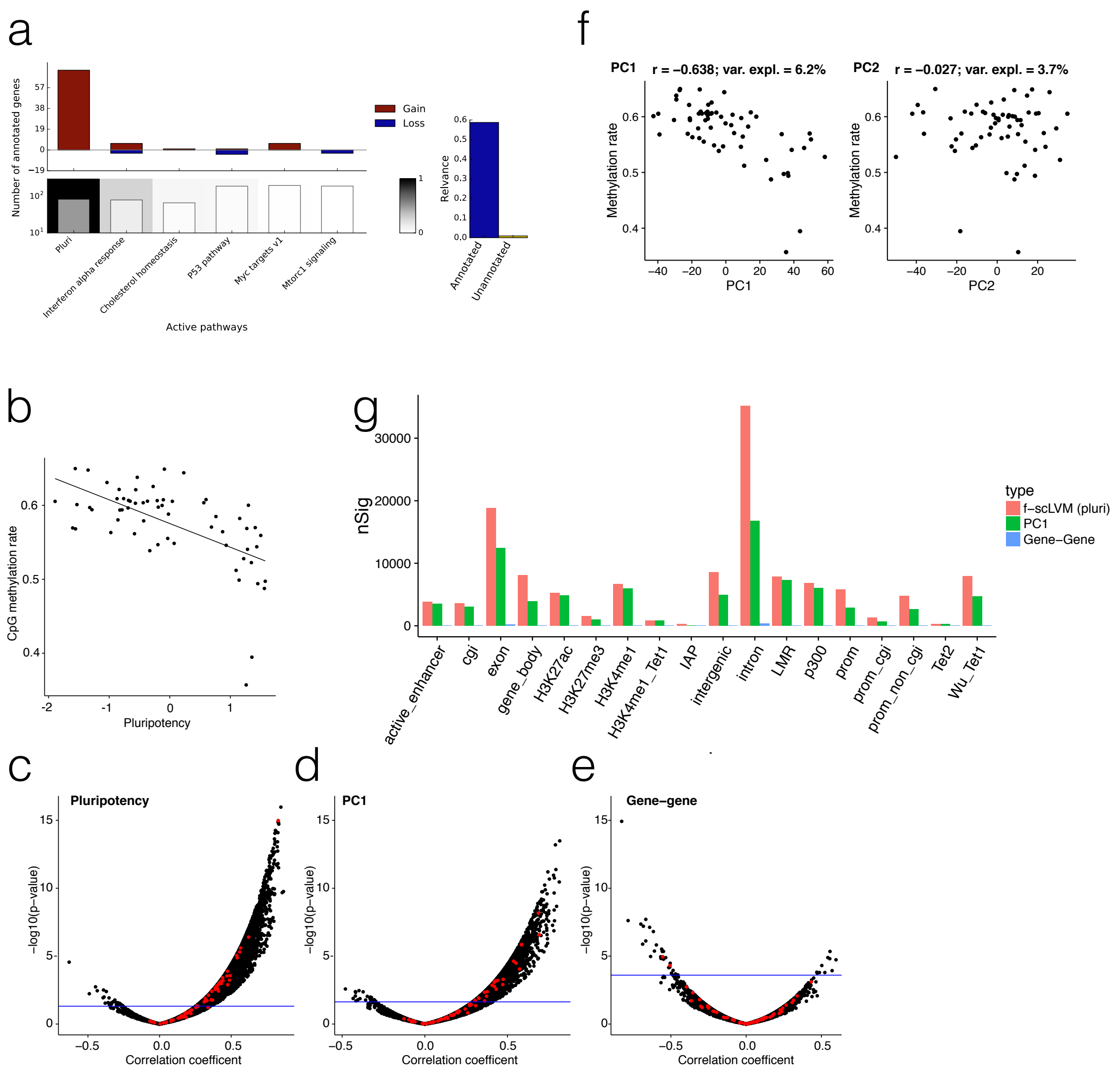

Figure SN2 | Application of f-scLVM to 61 mouse embryonic stem cells profiled using parallel DNA methylation & transcriptome sequencing. (a) Factor relevance of individual factors as determined by f-scLVM. **(b)** Scatter plot between the inferred pluripotency factor (Pluripotency) and genome-wide methylation rate in the same cells. The black solid line denotes a linear trend. **(c)** Genome-wide analysis of associations between gene-body methylation of individual genes and the inferred pluripotency factor, resulting in 8,124 significant associations (FDR<10%). Annotated pluripotency genes are marked in red. **(d,e)** Analogous association analysis when either considering the first PC on gene expression **(d)** or the expression level of the corresponding genes individually **(e)**, resulting in 3,907 and 43 significant associations respectively. The blue line corresponds to the 10% FDR threshold. **(f)** Association between methylation rate and PC1 and PC2 were weaker than those with the pluripotency factor. **(g)** Significant associations between methylation and expression for different genomic contexts. Applying f-scLVM consistently yielded the highest number of associations.

T-cells

We considered a dataset of differentiating T-cells, where the cell cycle is known to influence heterogeneity in gene expression [18]. The data were normalized as described previously in [18], resulting in 7,073 variable genes, which were used for analysis. We augmented the MSigDB gene sets with the set of 121 Th2 marker genes introduced in the primary publication [18]. f-scLVM was applied using the log-transformed normalized gene expression matrix for 81 cells. Again f-scLVM, identified factors with plausible annotations (Fig. SN3a), including processes related to the cell cycle (G2M Checkpoint and E2F targets) and to T-cell development (IL2/Stat5 signaling, Myc targets and Th2 genes). Reassuringly, the Th2 factor differentiated two previously identified subpopulations [18] of differentiating Th2 cells (Fig. SN3b).

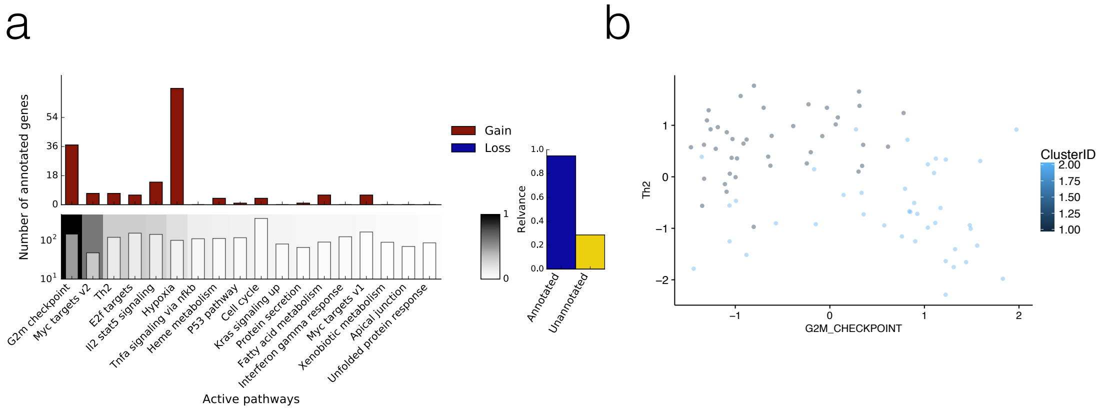

Figure SN3 | Application of f-scLVM to 81 Th2 cells. (a) Pathway factor relevance as identified by the f-scLVM model (using MSIGDB and an additional Th2 factor based on the gene set considered in Buettner et al., 2015[18]). The top ranked factors were the cell cycle (G2M checkpoints and E2F targets) and factors related to Th2 differentiation (Myc targets and Th2 differentiation). **(b)** Bivariate visualization of all cells using the inferred factors G2M checkpoint (cell cycle) and Th2 differentiation. The Th2 factor separated two cell populations that correspond to less differentiated cells (blue, GATA 3 low) and further differentiated cells (grey, GATA 3 high). These annotated differentiation states are taken from the analysis reported in Buettner et al., 2015 [18].

Pancreas data

We applied f-scLVM to 269 cells extracted from a human pancreas [19,20]. We filtered all non-low-quality cells from one individual (HP1502401) and used ERCC spike-ins to identify a set of 5,787 highly variable genes. The major drivers of variability were three hidden factors (sparse and dense), followed by Kras signaling and Complement System (Supp. Fig. 11a). The former is an important signaling pathway in the pancreas, both in health and disease [21], the latter known to be associated with ductal cells [22]. The Complement factor mainly separated ductal cells and the KRAS factor separated Delta and

Gamma cells (Supp. Fig. 11c). As for the Zeisel et al. data, the sparse hidden factors discriminated well known cell types (Supp. Fig. 11c). We quantified the discriminative power of the sparse hidden factors by training a naïve Bayes classifier on the active sparse hidden factors and computing the accuracy for predicting the correct cell type for each cell type (Supp. Fig. 11d).
We then assessed the ability of f-scLVM with Gaussian and hurdle noise model to discriminate known cell types with increasing dropout. To this end, we used a combination of the same two dropout mechanisms described in the section Simulation Study in the main text. To simulate a 20% and 30% dropout, we chose simulation parameters of 0.1 for the drop parameter (both settings) and a limit of detection of 4 and 8 in log-space, respectively. We ran 50 simulations of for each setting and compared the discriminative power of f-scLVM by again training a naïve Bayes classifier on the active sparse hidden factors for each noise model. The ability of f-scLVM to separate cell types based on sparse hidden factors degraded with increased dropout for both noise models; f-scLVM with dropout noise model consistently outperformed the Gaussian noise model with increased overall accuracies of up to 14% (for 30% dropout; Supp. Fig. 11d-f).

References

1. Vitek MP, Brown CM, Colton CA: **APOE genotype-specific differences in the innate immune response.** *Neurobiol Aging* 2009, **30:**1350-1360.
2. Nagarajan NA, Kronenberg M: **Invariant NKT cells amplify the innate immune response to lipopolysaccharide.** *J Immunol* 2007, **178:**2706-2713.
3. Rudy B, Fishell G, Lee S, Hjerling-Leffler J: **Three groups of interneurons account for nearly 100% of neocortical GABAergic neurons.** *Dev Neurobiol* 2011, **71:**45-61.
4. Macosko EZ, Basu A, Satija R, Nemesh J, Shekhar K, Goldman M, Tirosh I, Bialas AR, Kamitaki N, Martersteck EM, et al: **Highly Parallel Genome-wide Expression Profiling of Individual Cells Using Nanoliter Droplets.** *Cell* 2015, **161:**1202-1214.
5. Fujimoto Y, Tedder TF: **CD83: a regulatory molecule of the immune system with great potential for therapeutic application.** *J Med Dent Sci* 2006, **53:**85-91.
6. Murphy AC, Lalor SJ, Lynch MA, Mills KH: **Infiltration of Th1 and Th17 cells and activation of microglia in the CNS during the course of experimental autoimmune encephalomyelitis.** *Brain Behav Immun* 2010, **24:**641-651.
7. Sonobe Y, Yawata I, Kawanokuchi J, Takeuchi H, Mizuno T, Suzumura A: **Production of IL-27 and other IL-12 family cytokines by microglia and their subpopulations.** *Brain Res* 2005, **1040:**202-207.
8. Li Y, Chu N, Hu A, Gran B, Rostami A, Zhang GX: **Increased IL-23p19 expression in multiple sclerosis lesions and its induction in microglia.** *Brain* 2007, **130:**490-501.
9. Petropoulos S, Edsgard D, Reinius B, Deng Q, Panula SP, Codeluppi S, Reyes AP, Linnarsson S, Sandberg R, Lanner F: **Single-Cell RNA-Seq Reveals Lineage and X Chromosome Dynamics in Human Preimplantation Embryos.** *Cell* 2016, **167:**285.
10. Gardner DK, Larman MG, Thouas GA: **Sex-related physiology of the preimplantation embryo.** *Mol Hum Reprod* 2010, **16:**539-547.
11. Angermueller C, Clark SJ, Lee HJ, Macaulay IC, Mabel JT, Hu TX, Krueger F, Smallwood SA, Ponting CP, Voet T, et al: **Parallel single–cell bisulfite and RNA–sequencing link transcriptional and epigenetic heterogeneity.** *Nature Methods* 2016.
12. Kolodziejczyk AA, Kim JK, Tsang JC, Ilicic T, Henriksson J, Natarajan KN, Tuck AC, Gao X, Buhler M, Liu P, et al: **Single Cell RNA-Sequencing of Pluripotent States Unlocks Modular Transcriptional Variation.** *Cell Stem Cell* 2015, **17:**471-485.
13. Toyooka Y, Shimosato D, Murakami K, Takahashi K, Niwa H: **Identification and characterization of subpopulations in undifferentiated ES cell culture.** *Development* 2008, **135:**909-918.
14. Leitch HG, McEwen KR, Turp A, Encheva V, Carroll T, Grabole N, Mansfield W, Nashun B, Knezovich JG, Smith A, et al: **Naive pluripotency is associated with global DNA hypomethylation.** *Nat Struct Mol Biol* 2013, **20:**311-316.

15. Rakyan VK, Down TA, Balding DJ, Beck S: **Epigenome-wide association studies for common human diseases.** *Nat Rev Genet* 2011, **12:**529-541.
16. Brennecke P, Anders S, Kim JK, Kolodziejczyk AA, Zhang X, Proserpio V, Baying B, Benes V, Teichmann SA, Marioni JC, Heisler MG: **Accounting for technical noise in single-cell RNA-seq experiments.** *Nat Methods* 2013, **10:**1093-1095.
17. Stegle O, Teichmann SA, Marioni JC: **Computational and analytical challenges in single-cell transcriptomics.** *Nat Rev Genet* 2015, **16:**133-145.
18. Buettner F, Natarajan KN, Casale FP, Proserpio V, Scialdone A, Theis FJ, Teichmann SA, Marioni JC, Stegle O: **Computational analysis of cell-to-cell heterogeneity in single-cell RNA-sequencing data reveals hidden subpopulations of cells.** *Nat Biotechnol* 2015, **33:**155-160.
19. Segerstolpe Å, Palasantza A, Eliasson P, Andersson EM, Andréasson AC, Sun X, Picelli S, Sabirsh A, Clausen M, Bjursell MK, Smith DM: **Single-cell transcriptome profiling of human pancreatic islets in health and type 2 diabetes.** *Cell metabolism*. 2016, **24**(4):593-607.
20. Lun AT, Calero-Nieto FJ, Haim-Vilmovsky L, Gottgens B, Marioni JC: **Assessing The Reliability Of Spike-In Normalization For Analyses Of Single-Cell RNA Sequencing Data:** *bioRxiv*. 2017, 119784.
21. di Magliano MP, Logsdon CD: **Roles for KRAS in pancreatic tumor development and progression.** *Gastroenterology*. 2013, **144**(6):1220-9.
22. Andoh AK, Fujiyama YO, Sumiyoshi KE, Bamba TA: **Local secretion of complement C3 in the exocrine pancreas: ductal epithelial cells as a possible biosynthetic site.** *Gastroenterology*. 1996 ,**110**(6):1919-25.
